# WSES/GAIS/SIS-E/WSIS/AAST global clinical pathways for patients with intra-abdominal infections

**DOI:** 10.1186/s13017-021-00387-8

**Published:** 2021-09-25

**Authors:** Massimo Sartelli, Federico Coccolini, Yoram Kluger, Ervis Agastra, Fikri M. Abu-Zidan, Ashraf El Sayed Abbas, Luca Ansaloni, Abdulrashid Kayode Adesunkanmi, Boyko Atanasov, Goran Augustin, Miklosh Bala, Oussama Baraket, Suman Baral, Walter L. Biffl, Marja A. Boermeester, Marco Ceresoli, Elisabetta Cerutti, Osvaldo Chiara, Enrico Cicuttin, Massimo Chiarugi, Raul Coimbra, Elif Colak, Daniela Corsi, Francesco Cortese, Yunfeng Cui, Dimitris Damaskos, Nicola de’ Angelis, Samir Delibegovic, Zaza Demetrashvili, Belinda De Simone, Stijn W. de Jonge, Sameer Dhingra, Stefano Di Bella, Francesco Di Marzo, Salomone Di Saverio, Agron Dogjani, Therese M. Duane, Mushira Abdulaziz Enani, Paola Fugazzola, Joseph M. Galante, Mahir Gachabayov, Wagih Ghnnam, George Gkiokas, Carlos Augusto Gomes, Ewen A. Griffiths, Timothy C. Hardcastle, Andreas Hecker, Torsten Herzog, Syed Mohammad Umar Kabir, Aleksandar Karamarkovic, Vladimir Khokha, Peter K. Kim, Jae Il Kim, Andrew W. Kirkpatrick, Victor Kong, Renol M. Koshy, Igor A. Kryvoruchko, Kenji Inaba, Arda Isik, Katia Iskandar, Rao Ivatury, Francesco M. Labricciosa, Yeong Yeh Lee, Ari Leppäniemi, Andrey Litvin, Davide Luppi, Gustavo M. Machain, Ronald V. Maier, Athanasios Marinis, Cristina Marmorale, Sanjay Marwah, Cristian Mesina, Ernest E. Moore, Frederick A. Moore, Ionut Negoi, Iyiade Olaoye, Carlos A. Ordoñez, Mouaqit Ouadii, Andrew B. Peitzman, Gennaro Perrone, Manos Pikoulis, Tadeja Pintar, Giuseppe Pipitone, Mauro Podda, Kemal Raşa, Julival Ribeiro, Gabriel Rodrigues, Ines Rubio-Perez, Ibrahima Sall, Norio Sato, Robert G. Sawyer, Helmut Segovia Lohse, Gabriele Sganga, Vishal G. Shelat, Ian Stephens, Michael Sugrue, Antonio Tarasconi, Joel Noutakdie Tochie, Matti Tolonen, Gia Tomadze, Jan Ulrych, Andras Vereczkei, Bruno Viaggi, Chiara Gurioli, Claudio Casella, Leonardo Pagani, Gian Luca Baiocchi, Fausto Catena

**Affiliations:** 1 Department of Surgery Department of Surgery, Macerata Hospital, Macerata, Italy; 2 Department of General, Emergency and Trauma Surgery, Pisa University Hospital, Pisa, Italy; 3 Department of General Surgery, Rambam Health Care Campus, Haifa, Israel; 4 General Surgery Department, Regional Hospital of Durres, Durres, Albania; 5 Department of Surgery, College of Medicine and Health Sciences, UAE University, Al-Ain, United Arab Emirates; 6 Department of General and Emergency Surgery Faculty of Medicine, Mansoura University Hospital, Mansoura, Egypt; 7 Department of Surgery, Fondazione IRCCS Policlinico San Matteo, University of Pavia, Pavia, Italy; 8 Department of Surgery, Faculty of Clinical Sciences, College of Health Sciences, Obafemi Awolowo University, Osun State, Ile-Ife, Nigeria; 9 Department of General Surgery, Medical University of Plovdiv, UMHAT Eurohospital, Plovdiv, Bulgaria; 10 Department of Surgery, University Hospital Centre Zagreb, Zagreb, Croatia; 11 Trauma and Acute Care Surgery Unit, Hadassah Hebrew University Medical Center, Jerusalem, Israel; 12 Department of general surgery Bizerte hospital, Faculty of Medicine of Tunis, University Tunis El Manar, Tunis, Tunisia; 13 Department of Surgery, Lumbini Medical College and Teaching Hospital Ltd., Palpa, Tansen, Nepal; 14 Division of Trauma/Acute Care Surgery, Scripps Clinic Medical Group, La Jolla, CA USA; 15 Department of Surgery, Amsterdam University Medical Centers, location AMC, Amsterdam Gastroenterology Endocrinology Metabolism Research Institute, Amsterdam, The Netherlands; 16 Emergency and General Surgery Department, University of Milan-Bicocca, Milan, Italy; 17 Anesthesia and Transplant Surgical Intensive Care Unit, Ospedali Riuniti, Ancona, Italy; 18 Emergency Department, Niguarda Ca’Granda Hospital, Milan, Italy; 19 Riverside University Health System, CECORC Research Center, Loma Linda University, Loma Linda, USA; 20 Department of General Surgery, Health Sciences University, Samsun Training and Research Hospital, Samsun, Turkey; 21 General Direction, Area Vasta 3, ASUR Marche, Macerata, Italy; 22 Emergency Surgery Unit, San Filippo Neri’s Hospital, Rome, Italy; 23 Department of Surgery, Tianjin Nankai Hospital, Nankai Clinical School of Medicine, Tianjin Medical University, Tianjin, China; 24 Department of Surgery, Royal Infirmary of Edinburgh, Edinburgh, UK; 25 Minimally Invasive and Robotic Digestive Surgery Unit, Regional General Hospital F. Miulli, Bari, Italy; 26 Université Paris Est, UPEC, Creteil, France; 27 Department of Surgery, University Clinical Center of Tuzla, Tuzla, Bosnia and Herzegovina; 28 Department General Surgery, Kipshidze Central University Hospital, Tbilisi, Georgia; 29 Department of general, Digestive and Metabolic Minimally Invasive Surgery, Centre Hospitalier Intercommunal De Poissy/St Germain en Laye, Poissy, France; 30 Department of Pharmacy Practice, National Institute of Pharmaceutical Education and Research (NIPER), Hajipur, Bihar India; 31 Clinical Department of Medical, Surgical and Health sciences, Trieste University, Trieste, Italy; 32 General Surgery Unit, Sansepolcro Hospital, Arezzo, Italy; 33 Department of General Surgery, University of Insubria, University Hospital of Varese, ASST Sette Laghi, Regione Lombardia, Varese, Italy; 34 Department of Surgery, University Hospital of Trauma, Tirana, Albania; 35 Department of Surgery, Texas Health Resources, Fort Worth, TX USA; 36 Department of Medicine, Infectious Disease Division, King Fahad Medical City, Riyadh, Saudi Arabia; 37 Division of Trauma and Acute Care Surgery, Department of Surgery, University of California Davis, Sacramento, CA USA; 38 Department of Abdominal Surgery, Vladimir City Clinical Hospital of Emergency Medicine, Vladimir, Russia; 39 Department of General Surgery, Mansoura Faculty of Medicine, Mansoura University, Mansoura, Egypt; 40 Second Department of Surgery, Aretaieion University Hospital, National and Kapodistrian University of Athens, Athens, Greece; 41 Department of Surgery, Hospital Universitário Terezinha de Jesus, Faculdade de Ciências Médicas e da Saúde de Juiz de Fora, Juiz de Fora, Brazil; 42 Department of Upper GI Surgery, Queen Elizabeth Hospital, University Hospitals Birmingham NHS Foundation Trust, Birmingham, UK; 43 Trauma Service, Inkosi Albert Luthuli Central Hospital and Department of Surgery, Nelson R Mandela School of Clinical Medicine, Durban, South Africa; 44 Department of General and Thoracic Surgery, University Hospital Giessen, Giessen, Germany; 45 Department of Surgery, St. Josef Hospital, Ruhr University Bochum, Bochum, Germany; 46 Donegal Clinical Research Academy Emergency Surgery Outcome Project, Letterkenny University Hospital, Donegal, Ireland; 47 Surgical Clinic “Nikola Spasic”, Faculty of Medicine, University of Belgrade, Belgrade, Serbia; 48 Department of Emergency Surgery, City Hospital, Mozyr, Belarus; 49 Department of Surgery, Jacobi Medical Center, Albert Einstein College of Medicine, Bronx, NY USA; 50 Department of Surgery, Ilsan Paik Hospital, Inje University College of Medicine, Goyang, Republic of Korea; 51 General, Acute Care, Abdominal Wall Reconstruction, and Trauma Surgery, Foothills Medical Centre, Calgary, AB Canada; 52 Department of Surgery, Edendale Hospital, Pietermaritzburg, South Africa; 53 Department of General Surgery, University Hospital of Coventry & Warwickshire, Coventry, UK; 54 Department of Surgery #2, National Medical University, Kharkiv, Ukraine; 55 Division of Trauma and Surgical Critical Care, Department of Surgery, University of Southern California, Los Angeles, CA USA; 56 Department of General Surgery, School of Medicine, Istanbul Medeniyet University, Istanbul, Turkey; 57 Department of Pharmacy, Lebanese International University, Beirut, Lebanon; 58 Department of Surgery, Virginia Commonwealth University School of Medicine, Richmond, VA USA; 59 Global Alliance for Infections in Surgery, Macerata, Italy; 60 School of Medical Sciences, Universitiy Sains Malaysia, Kota Bharu, Kelantan Malaysia; 61 Abdominal Center, Helsinki University Hospital and University of Helsinki, Helsinki, Finland; 62 Department of Surgical Disciplines, Immanuel Kant Baltic Federal University, Regional Clinical Hospital, Kaliningrad, Russia; 63 Department of General and Emergency Surgery, ASMN, Reggio Emilia, Italy; 64 Department of Surgery, Universidad Nacional de Asuncion, Asuncion, Paraguay; 65 Department of Surgery, University of Washington, Seattle, WA USA; 66 First Department of Surgery, Tzaneion General Hospital, Piraeus, Greece; 67 Department of Surgery, Università Politecnica delle Marche, Ancona, Italy; 68 Department of Surgery, Post-Graduate Institute of Medical Sciences, Rohtak, India; 69 Second Surgical Clinic, Emergency Hospital of Craiova, Craiova, Romania; 70 Ernest E Moore Shock Trauma Center at Denver Health, Denver, USA; 71 Department of Surgery, Division of Acute Care Surgery, and Center for Sepsis and Critical Illness Research, University of Florida College of Medicine, Gainesville, FL USA; 72 Department of Surgery, Emergency Hospital of Bucharest, Bucharest, Romania; 73 Department of Surgery, University of Ilorin Teaching Hospital, Ilorin, Nigeria; 74 Division of Trauma and Acute Care Surgery, Fundacion Valle del Lili, Cali, Colombia; 75 Department of Surgery, Universidad del Valle, Cali, Colombia; 76 Department of Surgery, Hassan II University Hospital, Medical School of Fez, Sidi Mohamed Benabdellah University, Fez, Morocco; 77 Department of Surgery, University of Pittsburgh School of Medicine, UPMC-Presbyterian, Pittsburgh, USA; 78 Department of Emergency Surgery, Parma Maggiore Hospital, Parma, Italy; 79 3rd Department of Surgery, Attiko Hospital, MSc “Global Health-Disaster Medicine”, National and Kapodistrian University of Athens (NKUA), Athens, Greece; 80 Department of Surgery, UMC Ljubljana, Ljubljana, Slovenia; 81 National Institute for Infectious Diseases - INMI - Lazzaro Spallanzani IRCCS, Rome, Italy; 82 Department of General and Emergency Surgery, Cagliari University Hospital, Cagliari, Italy; 83 Department of Surgery, Anadolu Medical Center, Kocaeli, Turkey; 84 Infection Control, Hospital de Base, Brasília, DF Brazil; 85 Department of General Surgery, Kasturba Medical College & Hospital, Manipal Academy of Higher Education, Manipal, India; 86 General Surgery Department, Colorectal Surgery Unit, La Paz University Hospital, Madrid, Spain; 87 General Surgery Department, Military Teaching Hospital, Dakar, Senegal; 88 Department of Aeromedical Services for Emergency and Trauma Care, Ehime University Graduate School of Medicine, Ehime, Japan; 89 Department of Surgery, Western Michigan University School of Medicine, Kalamazoo, MI USA; 90 Department of Medical and Surgical Sciences, Emergency Surgery & Trauma, Fondazione Policlinico Universitario A. Gemelli IRCCS, Rome, Italy; 91 Department of General Surgery, Tan Tock Seng Hospital, Singapore, Singapore; 92 Department of Emergency medicine, Anesthesiology and critical care, Faculty of Medicine and Biomedical Sciences, University of Yaoundé I, Yaoundé, Cameroon; 93 Surgery Department, Tbilisi State Medical University, Tbilisi, Georgia; 94 First Department of Surgery, Department of Abdominal, Thoracic Surgery and Traumatology, First Faculty of Medicine, Charles University and General University Hospital, Prague, Czech Republic; 95 Department of Surgery, Clinical Center University of Pecs, Pecs, Hungary; 96 Department of Anesthesiology, Neuro Intensive Care Unit, Florence Careggi University Hospital, Florence, Italy; 97 Department of Surgery, Camerino Hospital, Macerata, Italy; 98 Department of Molecular and Translational Medicine, University of Brescia, Brescia, Italy; 99 Department of Infectious Diseases, Bolzano Hospital, Bolzano, Italy; 100 Department of Surgery, AAST Cremona, Cremona, Italy; 101 Department of Clinical and Experimental Sciences, University of Brescia, Brescia, Italy

**Keywords:** Intra-abdominal infections, Peritonitis, Sepsis

## Abstract

Intra-abdominal infections (IAIs) are common surgical emergencies and have been reported as major contributors to non-trauma deaths in hospitals worldwide. The cornerstones of effective treatment of IAIs include early recognition, adequate source control, appropriate antimicrobial therapy, and prompt physiologic stabilization using a critical care environment, combined with an optimal surgical approach. Together, the World Society of Emergency Surgery (WSES), the Global Alliance for Infections in Surgery (GAIS), the Surgical Infection Society-Europe (SIS-E), the World Surgical Infection Society (WSIS), and the American Association for the Surgery of Trauma (AAST) have jointly completed an international multi-society document in order to facilitate clinical management of patients with IAIs worldwide building evidence-based clinical pathways for the most common IAIs. An extensive non-systematic review was conducted using the PubMed and MEDLINE databases, limited to the English language. The resulting information was shared by an international task force from 46 countries with different clinical backgrounds. The aim of the document is to promote global standards of care in IAIs providing guidance to clinicians by describing reasonable approaches to the management of IAIs.

## Background

Intra-abdominal infections (IAIs) are common surgical emergencies and have been reported as major contributors to non-trauma deaths in emergency surgical units worldwide.

The cornerstone of effective treatment of IAIs includes early recognition, adequate source control, appropriate antimicrobial therapy, and prompt physiologic stabilization using intravenous fluid therapy in critically ill patients.

Results from published clinical trials often may not be representative of the true morbidity and mortality rates of such severe infections. Firstly, patients who have perforated appendicitis are usually over-represented in clinical trials. Secondly, patients with IAIs enrolled in clinical trials have often an increased likelihood of cure and survival. This is due to the fact that selective trial eligibility criteria usually exclude patients with comorbid diseases and other factors which are associated with death from IAI [1]. Affecting both high-income countries and low- and middle-income countries (LMICS), IAIs are a tremendous source of lost life, livelihood, and resources. In the WISS study [1] which enrolled all patients older than 18 years with complicated IAIs, the overall mortality rate was 9.2% (416/4533).

IAIs respect the principles of egalitarianism, as they remain a potential threat to the health of all humans of all age groups, race, and socioeconomics. Globally, the burden of IAIs is tremendous [2]. Patients with prolonged IAIs are more likely to present with severe metabolic compromise and exhaustion. This leads to prolonged intensive care unit (ICU) stays progressing into chronic critical illness and prolonged recovery with overall dismal long-term outcomes [3, 4]. Although it is still high, mortality after surgical sepsis has substantially decreased over the past 15 years as a result of early sepsis screening and reliable implementation of evidence-based ICU care [3]. Many patients who previously succumbed to early refractory shock and later multiple organ failure, now survive and develop a clinical trajectory of chronic critical illness, with a prolonged ICU course, high resource utilization, and persistent but manageable organ dysfunction. These patients have an underlying pathophysiologic syndrome of persistent inflammation, immunosuppression, and catabolism. Almost all are discharged to high resource post-discharge care facilities and are known to be associated with poor long-term outcomes.

A wide variety of organizations have published guidelines outlining the clinical management of IAIs [5–11]; however, there are no recently updated guidelines suitable for worldwide use.

In hospitals worldwide, non-acceptance of or non-compliance with or lack of access to evidence-based practices and guidelines in hospitals result in the overall poorer outcome of patients suffering from IAIs. Together, the World Society of Emergency Surgery (WSES), the Global Alliance for Infections in Surgery (GAIS), the Surgical Infection Society-Europe (SIS-E), The World Surgical Infection Society (WSIS), and the American Association for the Surgery of Trauma (AAST) have jointly completed an international multi-society document to promote global standards of care in IAIs providing guidance to clinicians by describing reasonable approaches to the management of IAIs.

An extensive non-systematic review was conducted using the PubMed and MEDLINE databases, limited to the English language. The resulting information was shared by an international task force from 46 countries worldwide with varying backgrounds.

## Principles of management

### Principles of diagnosis

Adequate detection and treatment are essential to minimize complications of IAIs [12, 13]. Diagnosis of IAIs is primarily clinical.

Patients with IAIs typically present with rapid-onset abdominal pain and signs of local and systemic inflammation (pain, tenderness, fever, increased white blood cell count, tachycardia, and/or tachypnea). Hypotension and signs of hypoperfusion such as oliguria, acute alteration of mental status, and lactic acidosis are indicative of ongoing organ failure.

Physical evaluation may limit the differential diagnoses to better direct decisions regarding a proper management plan including the selection of appropriate diagnostic testing, the need for initiation of antibiotic therapy, and whether emergent intervention is required [6].

The value of physical findings in the diagnostic work-up for IAIs has been studied in relation to acute appendicitis where signs and symptoms are helpful in diagnosing or excluding appendicitis [12, 13].

Inflammatory markers such as C-reactive protein (CRP) and procalcitonin (PCT) have been evaluated in the diagnosis of bacterial infection. CRP is an acute-phase protein promptly released during an inflammation. Since systemic bacterial infection is often associated with an inflammatory reaction, it represents an indirect marker of infection and inflammation [14].

Conversely, PCT rapidly increases in the presence of bacterial and fungal infections but not viral infections or noninfectious inflammation [14].

PCT has been evaluated in the management of IAIs, both for diagnosis and for guiding antibiotic therapy. However, it is important to take into account the limitations of biomarkers to ensure the appropriate use of them. Importantly, as with any diagnostic tool, PCT and CRP should be used embedded in clinical algorithms adapted to the type of infection and the clinical context and setting [15]. Moreover, PCT is released into the circulation when monocytic cells adhere and interact with parenchymal cells as an expression of a systemic response to infection that might not occur in localized infections such as abscesses, where PCT is not as useful [16]. Ultrasound (US) and computed tomography (CT) have been used over the last two decades to complete the clinical assessment of patients with IAIs. Although CT has higher sensitivity and specificity [17, 18], concerns about radiation exposure have recently prompted the reappraisal of the roles of sonography when performed by appropriately trained and accredited surgeons [19].

Proposals of staged algorithms using a step-up approach with CT performed after an inconclusive or negative US have been proposed in the setting of acute appendicitis and acute diverticulitis [20–23].

In order to identify an optimal imaging strategy for the accurate detection of urgent conditions in patients with acute abdominal pain, a multicenter diagnostic accuracy study using prospective data collection has been published [18]. In this study, CT led to the largest increase in accuracy after clinical evaluation, but a conditional strategy with CT after negative or inconclusive US resulted in the highest overall sensitivity, with only 6% missed urgent conditions, and the lowest overall exposure to radiation. The authors therefore recommended using US as the initial investigation in the diagnostic investigation of patients presenting with acute abdominal pain, with CT reserved for situations when US was negative or inconclusive.

Magnetic resonance imaging (MRI) is not routinely available in most hospitals in the emergency setting. Its use has been proposed in pregnant patients with abdominal pain when US is inconclusive [22, 23].

**Fig. 1:**
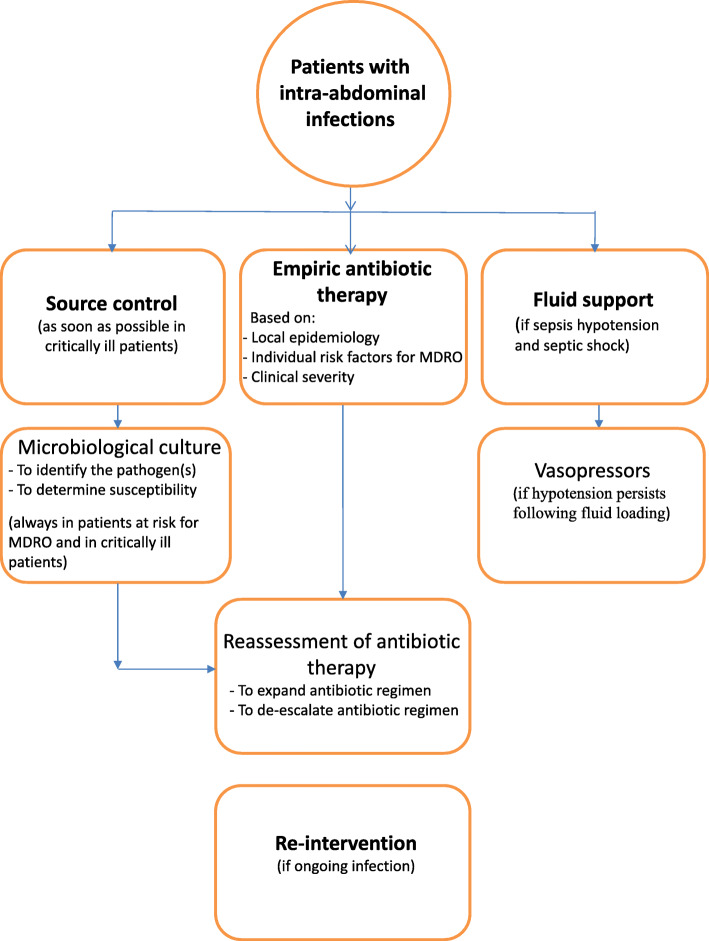
Principles of management of IAIs

Once the diagnosis is made, initial management of IAI includes an appropriate control of the source of the infection, adequate antibiotic therapy directed against the likely pathogens, and the prompt physiologic stabilization of the patient using intravenous fluid therapy (Fig. 1).

### Principles of source control

The term “source control” encompasses all those physical measures used to control a focus of invasive infection and to restore the optimal function of the affected area [24].

Intra-abdominal infections along with soft tissues infections are the sites where a source control is more feasible and more impactful.

Appropriate source control is of utmost importance in the management of IAIs. In these settings, appropriate source control can improve patients’ outcomes. Even though not definitively tested by randomized control trials, the magnitude of the increase in death and other adverse outcomes associated with inadequate source control makes it clear that it is of prime importance in treating most patients with IAIs. Moreover, an adequate source control can also shorten the course of antibiotic therapy. The impact of source control seems to be unrelated to the administration of appropriate antibiotics. Several studies found that both are independent predictors of mortality [25], but there is consensus that without adequate source control, antibiotic therapy may have little if any effect.

Source control assumes a comprehensive knowledge of biologic principles, the complexities of the infection response, the range of surgical and nonsurgical options, and a combination of therapeutic aggressiveness and judicious caution in the clinician charged with making the decision. Appropriate source-control intervention can rapidly alter the course of intra-abdominal infections to a more favorable direction, and suboptimal decision-making can change a difficult clinical challenge into a clinical burden [24].

The rules of source control are the following: (First) Time, Totalization, Technique, and (Second) Time.

The first time is related to the starting time of the treatment. Each hour in delaying represents a negative factor in the outcome.

The goal of the procedure is the totally (totalization) removing of any infective source and damaged tissues, opening spaces and compartment, evacuating pus or other fluids, deriving or resecting of any intestinal critical ischemic tracts also without immediate continuity restoration, and washing out the abdominal cavity. The surgical technique should be always correct and adequate.

The second time is related to the timing of further surgical control that may be either “on demand” or planned aimed to avoid a more dangerous surgical trauma in a critical patient.

The level of urgency of treatment is determined by the affected organ(s), the relative speed at which clinical symptoms progress and worsen, and the underlying physiological stability of the patient.

Surviving Sepsis Campaign Guidelines for Management of Sepsis and Septic Shock recommend that a specific anatomic diagnosis of infection requiring emergent source control be identified or excluded as rapidly as possible in patients with sepsis or septic shock, and that any required source control intervention be implemented as soon as medically and logistically practical after the diagnosis is made. Studies of septic patients undergoing source control for IAIs suggest that delays of only 3–6 h were associated with an increased mortality [25–27]. Although source control is the standard of care for most patients with IAIs, certain highly selected patients with a localized IAIs have been treated successfully with antibiotic therapy alone.

The control of the source of infection can be achieved using both operative and non-operative techniques. An operative intervention remains the most viable therapeutic strategy for managing surgical infections in critically ill patients [28].

The selection of a specific source control procedure for a patient should be predicated both on the characteristics of the infection and the patient, as well as the availability of technical expertise at the local institution.

Non-operative interventional procedures include percutaneous drainages of abscesses. Ultrasound-and CT-guided percutaneous drainage of abdominal and extraperitoneal abscesses in selected patients are safe and effective. The principal cause for failure of percutaneous drainage is the misdiagnosis of the magnitude, extent, complexity, and location of the abscess.

Surgery is the most important therapeutic measure to control surgical infections. In the setting of IAIs the primary objectives of surgical intervention include a) determining the cause of the infection, b) draining fluid collections, and c) controlling the origin of the infection. In patients with IAIs, surgical source control entails resection or suture of a diseased or perforated viscus (e.g. diverticular perforation, gastroduodenal perforation), removal of the infected organ (e.g. appendix, gallbladder), debridement of necrotic tissue, resection of ischemic bowel, and repair/resection of traumatic perforations with primary anastomosis or creation of stomas.

Traditionally, extensive lavage of the peritoneal cavity is used in the management of IAIs. Several recent series and one prospective trial of patients with perforated appendicitis likewise have found that aspiration and limited irrigation to remove gross contamination were as effective as lavage [29–31]. Surgical strategies following an initial emergency laparotomy include subsequent “re-laparotomy on demand” (when required by the patient’s clinical condition) as well as planned re-laparotomy in the 36–48-h post-operative period. An on-demand laparotomy is performed only when the patient deteriorates or fails to improve and only for those patients with CT findings indicating a clear benefit from additional surgery. Planned relaparotomies, on the other hand, are performed every 36–48 h for purposes of inspection, drainage, and peritoneal lavage of the abdominal cavity. The concept of a planned relaparotomy for severe peritonitis has been debated for over thirty years. Re-operations are performed every 48 h to reassess the peritoneal inflammatory process until the abdomen is free of ongoing peritonitis; then, the abdomen is closed. The advantages of the planned re-laparotomy approach are optimization of resource utilization and reduction of the potential risk for gastrointestinal fistulas and delayed hernias. The results of a clinical trial published in 2007 by Van Ruler et al. [32] investigating the differences between on-demand and planned re-laparotomy strategies in patients with severe peritonitis found few advantages for the planned re-laparotomy strategy; 232 patients with severe IAIs (116 on-demand and 116 planned) were randomized. Patients in the on-demand relaparotomy group did not have a significantly lower rate of adverse outcomes compared with patients in the planned relaparotomy group when the fascia was formally closed but did have a substantial reduction in relaparotomies, healthcare utilization, and medical costs. Patients in the on-demand group had shorter median ICU stays (7 versus 11 days; *p* = 0.001) and shorter median hospital stays (27 versus 35 days; *p* = 0.008). Direct medical costs per patient were reduced by 23% using the on-demand strategy.

The open abdomen (OA) may seem a viable option to some for treating physiologically deranged patients with ongoing sepsis, facilitating subsequent exploration and control of abdominal contents, and preventing abdominal compartment syndrome. The OA concept is closely linked to damage control surgery. However, the OA principle is the complete opposite of the on-demand relaparotomy strategy; the latter being a level 1 evidence proven beneficial strategy. Abdominal compartment syndrome in non-trauma patients can be mainly prevented by modern ICU techniques and fluid management. Deliberately leaving an abdomen open, which could otherwise be closed primarily, introduces risks of excessive fluid and electrolyte loss, undirected explorations, and intestinal fistula [33].

Every day an abdomen is left open, the risk of intestinal fistula increases. When closure is not possible at index laparotomy due to visceral edema, a negative pressure-based temporary closure device for progressive secondary fascial closure is used.

Open abdomen combined with negative pressure therapy is a different and more recent concept of abdominal sepsis treatment. In an open abdomen, the use of a (commercial) negative pressure device reduces mortality. Unclear is whether the benefits of this strategy are a reason to leave an abdomen open that could otherwise be closed. In order to define the role of OA with negative pressure therapy for improved biomediator clearance and mitigated systemic sepsis in patients with severe peritonitis the results of ongoing prospectively randomized trials, such as the COOL Trial, are needed [34].

Open abdomen combined with negative pressure therapy and fluid instillation is taking this therapeutic concept one step further, and results are promising [35, 36]. Severe complications including loss of the abdominal domain, fistula formation, and the development of giant incisional hernias may be observed when leaving the abdomen open without active closure device. The goal should be early and definitive closure of the abdomen, in order to reduce the complications associated with an open abdomen. Early definitive closure (within 7 days of the initial laparostomy) is the basis of preventing or reducing the risk of complications.

### Principles of antibiotic management

Antibiotics should be used after a treatable infection has been recognized or if there is a high degree of suspicion of an infection. The prolonged and inappropriate use of antibiotics appears a key factor in the rapid rise of antimicrobial resistance worldwide over the past decade. A rational and appropriate use of antibiotics is particularly important both to optimize quality clinical care and to reduce selection pressure on resistant pathogens.

In the setting of uncomplicated IAIs, such as uncomplicated appendicitis or cholecystitis, single doses have the same impact as multiple doses and post-operative antimicrobial therapy is not necessary if source control is adequate [9].

In the setting of complicated IAIs, a short course of antibiotic therapy after adequate source control is a reasonable option. The recent prospective trial by Sawyer et al. [37] demonstrated that in patients with complicated IAIs undergoing an adequate source control, the outcomes after approximately 4 days of fixed-duration antibiotic therapy were similar to those after a longer course of antibiotics that extended until after the resolution of physiological abnormalities.

Short-course antibiotics were demonstrated also in patients with post-operative IAIs [38].

A multicenter prospective randomized trial conducted in 21 French intensive care units (ICU) between May 2011 and February 2015 compared the efficacy and safety of 8-day versus 15-day antibiotic therapy in critically ill patients with post-operative IAIs. Patients treated for 8 days had a higher median number of antibiotic-free days than those treated for 15 days versus 12 days, respectively; *p* < 0.0001) (Wilcoxon rank difference 4.99 days [95% CI 2.99–6.00; *p* < 0.0001). Equivalence was established in terms of 45-day mortality (rate difference 0.038, 95% CI − 0.013 to 0.061). Treatments did not differ in terms of ICU and hospital length of stay, emergence of multi-drug-resistant (MDR) bacteria, or reoperation rate.

Short-course antibiotic therapy in critically ill ICU patients with post-operative IAIs reduced antibiotic exposure. Continuation of treatment until day 15 is not associated with any clinical benefit.

However, in patients with evidence of an ongoing infection, an individualized approach should be mandatory and the patient’s inflammatory response should be monitored regularly and decisions to continue, narrow, or stop antibiotic therapy must be made on the basis of clinician judgment and laboratory (such as CRP or PCT levels) investigations.

Patients who have ongoing signs of infection or systemic illness beyond 5–7 days of antibiotic treatment normally warrant a diagnostic investigation to determine whether additional surgical intervention or percutaneous drainage is necessary to address an ongoing uncontrolled source of infection or antibiotic treatment failure [39].

Initial antibiotic therapy for IAIs is typically empiric in nature because a patient with abdominal sepsis needs immediate treatment, and microbiological data (culture and susceptibility results) can require up to 48–72 h before they are available for a more detailed analysis.

Obtaining microbiological results from peritoneal fluid cultures from the site of infection has two advantages: (a) it provides an opportunity to expand the antimicrobial regimen if the initial choice was too narrow, and (b) it also allows de-escalation of antimicrobial therapy if the empirical regimen was too broad. Intraperitoneal specimens for microbiological evaluation from the site of infection are always recommended for patients with hospital-acquired IAIs or with community-acquired IAIs at risk for resistant pathogens and in critically ill patients. Obtaining cultures in all patients with IAIs for epidemiologic purposes if adequate resources are available can also allow aggregation and analysis of the data and the information can be used to guide institutional empiric antibiotic therapy policies. The choice of empiric antibiotic regimens in patients with IAI should be based on the local resistance epidemiology, the individual risk for infection by resistant pathogens, and the clinical condition of the patients [40].

Empiric antibiotic therapy for patients with IAI should include agents with activity against aerobic Gram-negative bacteria (e.g., Enterobacteriaceae), aerobic streptococci, and obligate enteric anaerobic organisms found in the gastrointestinal tract, although coverage of the latter may not be absolutely essential in patients with an upper gastrointestinal source of infection. Additional antibiotic agents, providing coverage of less common resistant or opportunistic pathogens such as *Candida* spp*.*, may be warranted in selected conditions. Generally, the most important factors in predicting the presence of resistant pathogens in IAIs is acquisition in a healthcare setting (particularly if the patient becomes infected in the ICU or has been hospitalized for more than 1 week), corticosteroid use, organ transplantation, baseline pulmonary or hepatic disease, and previous antimicrobial therapy [40].

In the last two decades, antimicrobial resistance has become a global threat to public health systems and some of the most common causes of misuse of antibiotics, and poor prevention and control with respect to infections. Particularly, infections caused by resistant gram-negative bacteria are becoming increasingly prevalent and now constitute a serious threat to public health worldwide because they are difficult to treat and are associated with high morbidity and mortality rates. Bacterium-producing carbapenemases, such as *K. pneumoniae*, are rapidly emerging as a major source of multidrug-resistant infections worldwide [41–44] and pose a serious threat in clinical situations where administration of effective empiric antibiotics is essential to prevent mortality following bacteremia and infections in immunocompromised patients. Non-fermenting gram-negative bacteria (*P. aeruginosa*, *Stenotrophomonas maltophilia*, *and Acinetobacter baumannii*) have exhibited alarming rates of increased resistance to a variety of antibiotics in health facilities worldwide. These species are intrinsically resistant to several drugs and could acquire additional resistance to other important antimicrobial agents [40].

In the context of IAIs, the main resistance problem is posed by extended-spectrum beta-lactamases (ESBL)-producing *Enterobacteriaceae*, which are alarmingly prevalent in nosocomial infections and frequently observed in community-acquired infections, albeit to a lesser extent [45, 46]. ESBL are enzymes capable of hydrolyzing and inactivating a wide variety of beta-lactams, including third-generation cephalosporins, penicillins, and aztreonam [47, 48].

Most ESBLs of clinical interest are encoded by genes located on plasmids. These plasmids may also carry genes encoding resistance to other multiple drug classes including aminoglycosides and fluoroquinolones.

The main risk factors for ESBL are:

- hospitalization for 48h within the last 90 days,
- broad-spectrum antibiotics for 5 days within the last 90 days,
- colonization by ESBL within 90 days.

Among Gram-positive bacteria, enterococci may play a significant role in IAIs. Some studies have demonstrated poor outcomes among patients with documented enterococcal infections, particularly in those with post-operative IAIs where enterococci coverage should be always considered [49, 50].

The acquisition of glycopeptide resistance by enterococci has seriously affected the treatment and control of these organisms. Affected patients usually have multiple and relevant co-morbidities, with prolonged hospital stay and received long courses of broad-spectrum antibiotics [50].

IAIs may be managed by either single or multiple antibiotic regimens. Beta-lactam/beta-lactamase inhibitor combinations, including, amoxicillin/clavulanate, ticarcillin/clavulanate, piperacillin/tazobactam, have an in vitro activity against Gram-positive, Gram-negative and anaerobic bacteria. Increasing rates of antimicrobial resistance to amoxicillin/clavulanate among *E. coli* and other Enterobacteriaceae worldwide, during the last decade, has compromised the clinical utility of this agent for empiric therapy of serious Gram-negative infections and therefore should be used based on local rates of resistance [40]. Broad-spectrum activity of piperacillin/tazobactam, including anti-pseudomonal and anaerobic coverage, still make it an attractive option in the management of severe IAIs.

Most isolates of *E. coli* and other Enterobacteriaceae remain susceptible to third-generation cephalosporins and in combination with metronidazole and may be options for empiric therapy of non-severe IAIs. Fluoroquinolones (FQ) have been widely used in the treatment of IAIs because of their excellent activity against aerobic Gram-negative bacteria and tissue penetration. In recent years, the resistance of *E. coli* to FQ has risen over time [40]. The worldwide increase in FQ resistance among *E. coli* and other Enterobacteriaceae has limited the use of FQ for empirical treatment of IAIs.

For decades, carbapenems have been the antibiotics of first choice for ESBLs. The best option for targeting ESBLs (although lacking coverage of P. aeruginosa) is ertapenem, a once-daily administered carbapenem that otherwise shares the activity of imipenem, meropenem, and doripenem against most species, including ESBL producing pathogens. Imipenem/cilastatin, meropenem, and doripenem provide coverage for Gram-negative non-fermenting bacteria (e.g. *Pseudomonas aeruginosa* and *Acinetobacter baumannii*). The use of carbapenems should be limited to preserve the activity of this class of antibiotics because of the concern of emerging carbapenem resistance [40].

Other options include aminoglycosides, particularly for suspected infections by Gram-negative bacteria in critically ill patients, and tigecycline especially when multidrug-resistant bacteria are suspected, although caution is advised for the latter, in the setting of bacteremia.

Because of their serious toxic side effects including nephrotoxicity and ototoxicity, some authors do not recommend aminoglycosides for the routine empiric treatment of IAIs [40]. Other authors have questioned the clinical importance of the toxic side-effects [8], and their decreased activity in acidic environment such as pus [9]. They may be best reserved for patients with allergies to beta-lactam agents [8, 9] or when used in combination with beta-lactams for the treatment of IAIs in critically ill patients [11]. In any case, this class of antibiotics remains an important option in the antibiotic armamentarium for combating Gram-negative bacteria and widening the spectrum of the empirical therapy when difficult to treat Gram-negative bacteria are suspected.

Tigecycline and eravacycline are viable treatment options, especially in empiric therapy, for complicated IAIs due to their favorable in vitro activity against anaerobic organisms, enterococci, several ESBL-producing and in association carbapenemase-producing Enterobacteriaceae, *A. baumannii*, and *Stenotrophomonas maltophilia* [40]. They do not feature in vitro activity against *P. aeruginosa* or *Proteus mirabilis*. Caution is always advised in its use for suspected bacteremia and healthcare-associated pneumonia [40]. Ceftolozone/tazobactam and ceftazidime/avibactam are new antibiotics that have been approved for the treatment of complicated IAI infections (in combination with metronidazole) including infection by Gram-negative bacteria, though their role as empirical therapy remains to be defined [40]. Ceftolozane/tazobactam is valuable for treating infections caused by multidrug-resistant Gram-negative bacteria in order to preserve carbapenems (it is active against ESBL but not against carbapenemases). Ceftazidime/avibactam has demonstrated consistent activity against KPC and OXA-48-producing organisms (it has no activity against metallo-beta-lactamase-producing bacteria) [40].

Table 1 presents antibiotics for treating patients with IAIs based upon susceptibility.

**Table 1:** Antibiotics for treating patients with IAIs based upon susceptibility. Use local antibiogram data for choosing optimal antibiotics in the target population

Antibiotic	Anaerobic coverage	*Pseudomonas* coverage	Non-resistant enterococci coverage	Enterobacteriaceae coverage	ESBL coverage
Amikacin	−	+	−	+	+/−
Amoxicillin/clavulanate	+	−	+	+/−^a^	−
Ceftazidime/avibactam	−	+^b^	−	+^c^	+
Ceftolozane/tazobactam	−	+^b^	−	+	+
Cefotaxime	−	−	−	+	−
Ceftazidime	−	+	−	+	−
Ceftriaxone	−	−	−	+	−
Ciprofloxacin	−	+	−	+/−^a^	−
Eravacycline	+	−	+	+^e^	+
Ertapenem	+	−	+/−	+	+
Imipenem-cilastatin	+	+	+^d^	+	+
Meropenem	+	+	+/−	+	+
Metronidazole	+	−	−	−	−
Piperacillin/tazobactam	+	+	+	+	+/−
Tigecycline	+	−	+	+^e^	+

^a^Increasing rates of antimicrobial resistance among Enterobacteriaceae worldwide

^b^Active against MDR *Pseudomonas aeruginosa* except metallo-beta-lactamases (MBL)-producing *Pseudomonas aeruginosa*

^c^Active against carbapenemase-producing *Klebsiella pneumoniae* except MBL-producing Enterobacteriaceae

^d^Imipenem/cilastatin is more active against ampicillin-susceptible enterococci than ertapenem, meropenem, and doripenem

^e^Not active against *Proteus*, *Morganella*, and *Providencia*

The epidemiologic profile of *Candida* spp*.* in the context of IAIs is incompletely defined. Its clinical presence is usually associated with poor prognosis. Empirical antifungal therapy for *Candida* spp*.* is typically not recommended for patients with community-acquired IAIs, with the notable exceptions of critically ill patients or immunocompromised patients (due to neutropenia or concurrent administration of immunosuppressive agents, such as glucocorticosteroids, chemotherapeutic agents, and immunomodulators) [9].

Recently, the Infectious Diseases Society of America (IDSA) guidelines for the treatment of invasive candidiasis were developed and addressed Candida peritonitis [51]. IDSA guidelines suggested considering empiric antifungal therapy for patients with clinical evidence of intra-abdominal infection and significant risk factors for candidiasis, including recent abdominal surgery, anastomotic leaks, or necrotizing pancreatitis, who are doing poorly despite treatment for bacterial infections.

An ineffective or otherwise inadequate antibiotic regimen is one of the variables more strongly associated with unfavorable outcomes in critically ill patients. Broader empiric antibiotic therapy should be started as soon as possible in patients with organ dysfunction and septic shock, reassessing the antibiotic regimen once results of microbiological cultures are obtained.

Antibiotic de-escalation has been associated with lower mortality rates in ICU patients and is now considered a key practice for antimicrobial stewardship purposes.

Montravers et al. [52] valued the characteristics and outcomes of anti-infective de-escalation during healthcare-associated IAIs (HA-IAIs). They demonstrated that de-escalation is a feasible option in patients with polymicrobial infections such as HA-IAIs. However, MDR non-fermenting Gram-negative bacteria can limit its implementation in the setting of IAIs.

Moreover, maintaining antibiotic therapy for a long period may be a critical factor in developing extra-abdominal infections [53].

For patients with sepsis or septic shock, early and properly administered empirical antimicrobial therapy can have a significant impact on the outcome, independent of the anatomical site of infection. The Surviving Sepsis Campaign guidelines for the management of sepsis and septic shock [54] recommend intravenously administered antibiotics within the first hour of onset of sepsis and septic shock and the use of broad-spectrum agents with adequate penetration of the presumed site of infection. Additionally, the employed antibiotic regimen should be reassessed daily in order to optimize efficacy, prevent toxicity, minimize cost, and reduce selection pressures favoring resistant strains [7].

The antibiotic dosing regimen should be established depending on host factors and properties of antibiotic agents. The achievement of appropriate target site concentrations of antibiotics is essential to eradicate the relevant pathogen. Suboptimal target site concentrations may have important clinical implications and may explain therapeutic failures, in particular for bacteria for which in vitro minimum inhibitory concentrations (MICs) are high. Antibiotics typically need to reach a site of action outside the plasma. This requires the drug to pass through the capillary membranes. Both the disease state and drug-related factors can contribute to differential tissue distribution [55]. In the event of abdominal sepsis, clinicians must be aware that drug pharmacokinetics may be altered significantly in critically ill patients due to the pathophysiology of sepsis. The “dilution effect,” also known as the “third spacing phenomenon,” is very important for hydrophilic agents. Higher than standard loading doses (LD)s of hydrophilic agents such as beta-lactams, aminoglycosides, and glycopeptides should be administered to ensure optimal exposure at the infection site, maintaining a therapeutic threshold that withstands the effects of renal function [40]. For lipophilic antibiotics such as fluoroquinolones and tetracyclines, the “dilution effect” in extracellular fluids may be mitigated during severe sepsis by the rapid redistribution of drugs to the interstitium from the intracellular compartment. Unlike observations of subtherapeutic administration of standard-dose hydrophilic antimicrobials, standard dosages of lipophilic antibiotics are often sufficient to ensure adequate loading, even in patients with sepsis or septic shock.

Once an appropriate initial loading dose is achieved, the antibiotic regimen should be reassessed, at least daily, because pathophysiological changes may significantly affect drug availability in the critically ill patients. Lower than standard dosages of renally excreted drugs must be administered in the presence of impaired renal function, while higher than standard dosages of renally excreted drugs may be needed for optimal activity in patients with glomerular hyperfiltration [40]. It should be noted that in critically ill patients, plasma creatinine is an unreliable marker of renal function.

Knowledge of the pharmacokinetic and pharmacodynamic antibiotic properties of each drug including (inhibition of growth, rate and extent of bactericidal action, and post-antibiotic effect) may provide a more rational determination of optimal dosing regimens in terms of the dose and the dosing interval. Optimal use of the pharmacokinetic/pharmacodynamic properties of antibiotics is important for obtaining good clinical outcomes and reduction of resistance. Dosing frequency is related to the concept of time-dependent versus concentration-dependent killing. Beta-lactams exhibit time-dependent activity and exert optimal bactericidal activity when drug concentrations are maintained above the MIC [40]. Therefore, it is important that the serum concentration exceeds the MIC for the appropriate duration of the dosing interval for the antibiotic and the organism. Higher frequency dosing, prolonged infusions, and continuous infusions have been utilized to achieve this effect. Basing on pharmacokinetics/pharmacodynamics principles the traditional intermittent dosing of each agent may be replaced with prolonged infusions of certain beta-lactam antibiotics especially in those critically ill patients with infections caused by Gram-negative bacilli that have elevated but susceptible MICs to the chosen agent [55].

In contrast, antibiotics such as aminoglycosides exhibit concentration-dependent activity and should be administered in a once-daily manner (or with the least possible number of daily administrations) in order to achieve high peak plasma concentrations. With these agents, the peak serum concentration, and not the time the concentration remains above the MIC, is more closely associated with efficacy. In terms of toxicity, aminoglycosides nephrotoxicity is caused by a direct effect on the renal cortex and its uptake saturation [55]. Thus, an extended interval dosing strategy reduces the renal cortex exposure to aminoglycosides and reduces the risk of nephrotoxicity [40].

In 2017, the Global Alliance for Infections in Surgery published a global declaration on the appropriate use of antimicrobial agents across the surgical pathway and reported the following 16 principles of appropriate antibiotic therapy across the surgical pathways [56]:

1. The source of infection should always be identified and controlled as soon as possible.
2. Antibiotic empiric therapy should be initiated after a treatable surgical infection has been recognized, because microbiologic data (culture and susceptibility results) may not be available for up to 48–72 h to guide targeted therapy.
3. In critically ill patients, empiric broad-spectrum therapy to cover the most likely pathogens should be initiated as soon as possible after a surgical infection has been recognized. Empiric antimicrobial therapy should be narrowed once culture and susceptibility results are available and adequate clinical improvement is noted.
4. Empiric therapy should be chosen on the basis of local epidemiology, individual patient risk factors for MDR bacteria and *Candida* spp., clinical severity, and infection source.
5. Specimens for microbiologic evaluation from the site of infection are always recommended for patients with hospital-acquired or with community-acquired infections at risk for resistant pathogens (e.g., previous antimicrobial therapy, previous infection or colonization with a MDR, XDR, and PDR pathogen) and in critically ill patients. Blood cultures should be performed before the administration of antibiotic agents in critically ill patients.
6. The antibiotic dose should be optimized to ensure that PK-PD targets are achieved. This involves prescribing an adequate dose, according to the most appropriate and right method and schedule to maximize the probability of target attainment.
7. The appropriateness and need for antimicrobial treatment should be re-assessed daily.
8. Once source control is established, short courses of antibiotic therapy are as effective as longer courses regardless of signs of inflammation.
9. Intra-abdominal infection—4 days are as effective as 8 days in moderately ill patients
10. Blood stream infection—5 to 7 days are as effective as 7 to 21 days for most patients
11. Ventilator-associated pneumonia—8 days are as effective as 15 days.
12. Failure of antibiotic therapy in patients having continued evidence of active infection may require a reoperation for a second source control intervention.
13. Biomarkers such as procalcitonin may be useful to guide the duration and cessation of antibiotic therapy in critically ill patients.
14. Clinicians with advanced training and clinical experience in surgical infections should be included in the care of patients with severe infections.
15. The infection prevention and control measures combined with antimicrobial stewardship programs should be implemented in surgical departments. These interventions and programs require regular, systematic monitoring to assess compliance and efficacy.
16. Monitoring of antibiotic consumption should be implemented and feedback provided to all ASP team members regularly (e.g., every 3 to 6 months) along with resistance surveillance data and outcome measures.

### Principles of sepsis management

Sepsis is a complex, multifactorial syndrome which can evolve into conditions of varying severity. If left untreated, it may lead to the functional impairment of one or more vital organs or systems.

The data from the WISS study showed that mortality in patients with complicated IAIs was significantly affected by the clinical conditions. Sepsis status was no sepsis 1.2%, sepsis only 4.4%, severe sepsis 27.8%, and septic shock 67.8% [1].

IAIs accounted for the second origin of septic shock (after pulmonary sepsis) affecting 21.8% of 10,069 patients admitted into the intensive care units in a multinational study conducted in Europe, the USA, Asia, Africa, and the Oceania region in 2012 [57].

The Third International Consensus Definitions for Sepsis and Septic Shock (Sepsis-3) [58] has updated previous classifications [58, 59]. The pathophysiology of sepsis takes origin from the outer membrane components of both Gram-negative organisms (lipopolysaccharide [LPS], lipid A, endotoxin) and Gram-positive organisms (lipoteichoic acid, peptidoglycan) [60]. These outer membrane components are able to bind to the CD14 receptor on the surface of monocytes. By virtue of the recently described toll-like receptors, a signal is then transmitted to the cell, leading to the eventual production of the proinflammatory cytokines, including tumor necrosis factor (TNF), interleukin 1 (IL-1), IL-6, IL-8, and gamma interferon (IFN-gamma), as well as other inflammatory mediators such as prostaglandins, leukotrienes, platelet-activating factor, and nitrogen and oxygen intermediates.

Most of these immunological mediators present multiple biologic effects, play a critical role in inflammation and immune responses, and have been recognized as key mediators in the pathogenesis of infectious diseases, particularly in the pathophysiologic alterations observed in endotoxic shock. As a result of the vicious cycle of inflammation, cardiovascular insufficiency and multiple organ failure occur and often lead to death.

Sepsis is now defined as life-threatening organ dysfunction caused by a dysregulated host response to infection. Organ dysfunction can be represented by an increase in the Sequential (sepsis-related) Organ Failure Assessment (SOFA) score of 2 points or more. Septic shock should be defined as a subset of sepsis and should be clinically identified by vasopressor requirement to maintain a mean arterial pressure of 65 mm Hg or greater and serum lactate level greater than 2 mmol/L (> 18 mg/dL) in the absence of hypovolemia. The definition of severe sepsis is now superfluous. The new definition of sepsis suggests that patients with at least 2 of these 3 clinical variables: Glasgow Coma Scale score of 13 or less, systolic blood pressure of 100 mm Hg or less, and respiratory rate 22/min or greater (quick SOFA—qSOFA) may be prone to a poor outcome typical of sepsis and patients with positive qSOFA should be clinically characterized as septic by SOFA score (Table 2). The SOFA score was proposed in 1996 by the Working Group on Sepsis-Related Problems of the European Society of Intensive Care Medicine [61] to objectively describe the degree of organ dysfunction over time and to evaluate morbidity in the intensive care unit (ICU) patients with sepsis. It was demonstrated to be a good indicator of prognosis in critically ill patients during the first few days of ICU admission [62].

**Table 2:** SOFA score

	SOFA score
PaO_2_/FiO_2_ (mmHg)	
<400	1
<300	2
<200 and mechanicallyventilated	3
<100 and mechanicallyventilated	4
Glasgow coma scale	
13–14	1
10–12	2
6–9	3
<6	4
Mean arterial pressure OR administration of vasopressors required	
MAP <70 mm/Hg	1
dop ≤5 or dob (any dose)	2
dop >5 OR epi ≤0.1 OR nor ≤0.1	3
dop >15 OR epi >0.1 OR nor >0.1	4
Bilirubin (mg/dl)	
1.2–1.9	1
2.0–5.9	2
6.0–11.9	3
>12.0	4
Platelets × 10^3^/μl	
<150	1
<100	2
<50	3
<20	4
Creatinine (mg/dl)	
1.2–1.9	1
2.0–3.4	2
3.5–4.9	3
>5.	4

One of the most likely explanations for the high morbidity and mortality rates associated with sepsis is the development of cardiovascular insufficiency, which can lead to global tissue hypoxia. In sepsis, the early hemodynamic profile is characterized by hypovolemia, vaso-regulatory dysfunction, and myocardial depression. Increased capillary leakage and venous capacitance ultimately result in decreased venous return to the heart. Additionally, cytokines released during the patient’s immune response may trigger further myocardial depression. These hemodynamic alterations associated with the early stages of sepsis are often accompanied by an increase in systemic oxygen demand and impaired oxygen delivery, thereby inducing global tissue hypoxia. Global tissue hypoxia may overstimulate endothelial cell activity, which can subsequently contribute to the systemic inflammatory cascade characteristic of sepsis.

Fluid therapy to improve microvascular blood flow and increase cardiac output is an essential part of the treatment of patients with sepsis. Crystalloid solutions should be the first choice because they are well tolerated and cheap. They should be infused rapidly to induce a quick response but not so fast that an artificial stress response develops. They should be interrupted when no improvement of tissue perfusion occurs in response to volume loading. Basal lung crepitations may indicate fluid overload or impaired cardiac function. Recently, measuring inferior vena cava diameter by US was suggested as a novel outcome measure to guide this resuscitative approach [64].

An early identification of the septic state and prompt administration of intravenous fluids are mandatory. However, initial resuscitation should no longer be based on a predetermined protocol but on clinical endpoints. Hypotension is the most common indicator of inadequate perfusion.

Particularly in patients with abdominal sepsis, requiring urgent surgical intervention, overly aggressive fluid resuscitation may increase intra-abdominal pressure and worsen the inflammatory response, which is associated with a high risk of complications. In patients with septic shock, fluid infusion during resuscitation may lead to bowel edema and forced closure of the abdominal wall may cause intra-abdominal hypertension and abdominal compartment syndrome which can affect pulmonary, cardiovascular, renal, splanchnic, and central nervous system physiology causing significant morbidity and mortality.

Vasopressor agents should be administered to restore organ perfusion if fluid resuscitation fails to optimize blood flow and if hypotension persists following adequate fluid loading. These agents should be globally available. Vasopressor and inotropic agents have increasingly become a therapeutic cornerstone in the management of sepsis. They have excitatory and inhibitory actions on the heart and vascular smooth muscle, as well as important metabolic, central nervous system, and presynaptic autonomic nervous system effects. The most common catecholamine-active medications are phenylephrine, norepinephrine, and epinephrine. Each of these three medications has varying activity on the alpha and beta receptors. Alpha receptors are peripheral vasoconstrictors to increase SVR. Beta-1 receptors have mostly positive chronotropic (heart rate) and inotropic (contractility) effects on the heart. Beta-2 receptors act as vasodilators in many organ systems.

The optimal timing of vasopressors relative to fluid infusion has been debated. A large multi-center retrospective analysis of 2849 patients with septic shock found that mortality was lowest when vasopressors were delayed by 1 h and infused from hours 1 to 6 following onset of shock [65].

Norepinephrine is now the first-line vasopressor agent used to correct hypotension in the event of septic shock [54]. Norepinephrine is more efficacious than dopamine and may be more effective for reversing hypotension in patients with septic shock [54]. Norepinephrine has mixed alpha-1 and beta activity (beta-1 greater than beta-2), with slightly more alpha-1 activity compared to beta activity. This leads to a more significant increase in blood pressure than increased heart rate. Blood pressure, mean arterial pressure, systemic vascular resistance, and cardiac output are increased with norepinephrine.

Epinephrine has essentially equivocal activity on alpha-1 and beta receptors. Vasopressin acts on V-1 receptors to stimulate smooth muscle contraction of the vessels as well as V-2 receptors in the kidneys as an anti-diuretic. There are no inotropic or chronotropic effects. Only blood pressure and systemic vascular resistance are increased with vasopressin.

Vasopressin levels in septic shock have been reported to be lower than anticipated for a shock state. Low doses of vasopressin may be effective in raising blood pressure in those patients refractory to other vasopressors and may have other potential physiologic benefits [54].

Dopamine may cause more tachycardia and may be more arrhythmogenic than norepinephrine, and as an alternative vasopressor agent to norepinephrine, it should be used only in patients with low risk of tachyarrhythmias and absolute or relative bradycardia [66].

Dobutamine is an inotropic agent used to treat septic shock patients increasing cardiac output, stroke index, and oxygen delivery (DO2). It has been suggested to be administered to pre-existing vasopressor therapy in the presence of myocardial dysfunction, defined as elevated cardiac filling pressures and low cardiac output. However, dobutamine increases DO2 to supranormal values and in critically ill patients frequently leads to a worsening of the hypotension due to its peripheral vasodilation, it has raised serious questions regarding its safety in the treatment of septic shock. Because dobutamine provides direct stimulation of the β-1 adrenergic receptors, it is recognized as more problematic with regard to tachycardia and arrhythmia.

In LMICs it may be acceptable to use adrenaline infusions as the inotrope of choice, given it is readily available, cheap, and has been shown to be equivalent to noradrenaline for its vasopressor effects in septic shock but with a significant risk of tachycardia and tachyarrythmias [67].

Increased global availability of vasopressors together with a better understanding of their indications, pharmacodynamics, and important adverse effects is mandatory to fight sepsis worldwide.

Table 3 presents both the receptor activity and the dosage of the most common vasopressors.

**Table 3:** Receptor activity and dosage of the most common vasopressors

Vasopressor	Receptor activity	Dose (all intravenous)
Norepinephrine	α1 > β1, β2	5–100 μg/min
Epinephrine	α1 > β1, β2 More β1 than NE	5–60 μg/kg min
Phenylephrine	α1	50–100 μg bolus 0.1–1.5 μg/kg/min
Dopamine	DA1, DA2	1–5 μg/kg/min “low dose” 5–15 μg/kg/min moderate dose 20–50 μg/kg min high dose
Vasopressin	AVPR1a, AVPR1b, AVPR2	0.01–0.04 U/min

The use of corticosteroids has been debated in recent years. The Surviving Sepsis Campaign guidelines [68] suggest against using IV hydrocortisone to treat septic shock patients if adequate fluid resuscitation and vasopressor therapy are able to restore hemodynamic stability. If this is not achievable, the guidelines suggest IV hydrocortisone at a dose of 200 mg per day.

The “sepsis bundle” has been central to the implementation of the Surviving Sepsis Campaign from the first publication of its evidence-based guidelines in 2004 through subsequent editions [68].

The Surviving Sepsis Campaign Bundle 2018 update reported the following 5 “hour-1 bundles,” for improving outcomes in patients with sepsis and septic shock [54].

- Measure lactate level. Re-measure if initial lactate is > 2 mmol/L
- Obtain blood cultures prior to administration of antibiotics
- Administer broad-spectrum antibiotics
- Rapidly administer 30 ml/kg crystalloid for hypotension or lactate ≥ 4 mmol/L
- Apply vasopressors if the patient is hypotensive during or after fluid resuscitation to maintain MAP ≥ 65 mm Hg

## Acute appendicitis

Acute appendicitis (AA) is both the most common general surgery emergency as well as the most common cause of IAIs worldwide [69–71]. The WISS study [1] confirmed AA as the most frequent cause of intra-abdominal infections and demonstrated that around one-third of the cases were deemed complicated.

Interestingly, the incidence of acute appendicitis varies: it is generally thought to be low rate in sub-Saharan Africa and in many regions of Asia and Latin America. This condition once thought to be rare in many regions of the world appears to be increasing in many urban centers and also in LMICs, perhaps due to changes in lifestyle and diet [71]. However, the true incidence of AA in many areas of the world is unknown due to poor medical record keeping and unreliable population census.

In 2020, the World Society of Emergency Surgery updated the guidelines for the diagnosis and treatment of acute appendicitis [69].

The natural history of appendicitis has been described in two disease types: (1) uncomplicated acute appendicitis, and (2) complicated appendicitis, according to their macroscopic and microscopic appearance and clinical relevance. The high morbidity and occasional mortality associated with acute appendicitis are mostly related to the delay in the presentation by patients or delay in diagnosis by the clinician. These delays may result in complications like gangrene, perforation, appendiceal mass, and generalized peritonitis, all of which would prolong hospital stay and increase the cost of treatment. The rate of perforation varies from 16 to 40%, with a higher frequency occurring in younger age groups (40–57%) and in patients older than 50 years (55–70%) [72].

### Diagnosis

While the clinical diagnosis of acute appendicitis may be straightforward in patients who present with classic signs and symptoms, atypical presentations may result in diagnostic confusion and delay in treatment and are known as the “great mimic” for many intra-abdominal diseases. Moreover, a classical presentation of acute appendicitis [combination of migration of pain to the right lower quadrant (LRQ) and tenderness in the RLQ] occurs rarely in patients with suspected appendicitis. The diagnosis of appendicitis can be challenging even in the most experienced hands and is predominantly clinical.

#### Clinical signs and symptoms

- Abdominal pain: it usually has a gradual onset and increases intensity over time. It is usually relieved in the supine position and aggravated by coughing or abdominal movements. Typically, there may be a short history (1 to 3 days) of migration of the pain from the peri-umbilical region to the RLQ.
- Nausea and/or vomiting soon after abdominal pain begins
- Fever
- Tenderness localized in the RLQ (often in complicated acute appendicitis)

#### Laboratory markers

- Increased white blood cell count
- Leucocyte shift to left (>75%)
- Increased C-reactive protein) useful in predicting the risk of complicated acute appendicitis

#### Scores

Unfortunately, the clinical presentation of AA is often inconsistent. While the clinical diagnosis may be clear in patients presenting with classic signs and symptoms, atypical presentations may result in the delay in treatment. Therefore, diagnostic scoring systems have been described with the aim to provide clinical probabilities that a patient has acute appendicitis. The development of these scores may contribute to diagnosis and by easily applying clinical criteria and simple laboratory tests, a score estimating the probability of diagnosis may be attributed to the patient. The most common used scores are the Alvarado score (Table 4), the Andersson appendicitis inflammatory response (AIR) (Table 5), and the Adult appendicitis score (AAS) (Table 6).

**Table 4:** Alvarado score

Diagnostic criteria	Value
Pain migration to RLQ	1
Anorexia	1
Nausea–vomiting	1
Tenderness in RLQ	2
Cough/percussion tenderness	2
Elevated temperature ≥ 37.3 °C	1
Leucocytosis (> 10 × 103/L)	2
Leucocyte left shift (> 75%)	1
**Total score**	**10**

A score 0–4 indicates low probability of acute appendicitis

A score > 5 indicates probable appendicitis

A score > 8 indicates very probable appendicitis

**Table 5:** Andersson appendicitis inflammatory response (AIR)

Diagnostic criteria	Value
Pain or tenderness in right lower quadrant	1
Vomiting	1
Rebound tenderness or guarding	
Light	1
Moderate	2
Severe	3
WBC Count (> 75%)	
10–14.9 × 109/1	1
≥ 15.0 × 109/1	2
Segmented neutrophils	
70–84%	1
≥ 85%	2
CRP mg/l	
10–49	1
≥ 50	2
Body temperature ≥ 38.5 °C	1

A score of 0-4 indicates low probability of acute appendicitis

A score 5–8 indicates active observation with re-scoring/ imaging or diagnostic laparoscopy according to local practice

A score of 9–12 indicates high probability of acute appendicitis

**Table 6:** Adult Appendicitis Score (AAS)

Symptoms and findings	Score
Pain in RLQ	2
Pain relocation	2
RLQ tenderness	
Women, age 16–49	1
All other patients	3
Guarding	
Mild	2
Moderate or severe	4
Laboratory tests	
Blood leukocyte count (× 109)	
≥ 7.2 and < 10.9	1
≥ 10.9 and < 14.0	2
≥ 14.0	3
Proportion of neutrophils (%)	
≥ 62 and < 75	2
≥ 75 and < 83	3
≥ 83	4
CRP (mg/l), symptoms < 24 h	
≥ 4 and < 11	2
≥ 11and < 25	3
≥ 25 and < 83	5
≥ 83	1
CRP (mg/l), symptoms > 24 h	
≥ 12 and < 53	2
≥ 53 and < 152	2
≥ 152	1

*RLQ* the right lower abdominal quadrant

Score ≤ 10 low risk of appendicitis

Score 11–15 intermediate risk of appendicitis

Score ≥ 16 high risk of appendicitis

Although the Alvarado score is not sufficiently specific in diagnosing AA, a cutoff score of < 5 is sufficiently sensitive to exclude AA (sensitivity of 99%). The Alvarado score could, therefore, be used to reduce emergency department length of stay and radiation exposure in patients with suspected AA [73].

The Alvarado score is not able to differentiate complicated from uncomplicated AA in elderly patients [74]. Several attempts have been made to distinguish between uncomplicated and complicated forms on a clinical basis, but to date, no accessible routine severity index for acute appendicitis is available.

The RCT by Andersson et al. demonstrated that, in low-risk patients, the use of an AIR (Appendicitis Inflammatory Response) score-based algorithm resulted in less imaging (19.2% versus 34.5%, *p* < 0.001), fewer admissions (29.5% versus 42.8%, *p* < 0.001), fewer negative explorations (1.6% versus 3.2%, *p* = 0.030), and fewer surgical operations for non-perforated AA (6.8% versus 9.7%, *p* = 0.034). Intermediate-risk patients randomized to the imaging and observation strategies had the same proportion of negative appendectomies (6.4% versus 6.7%, *p* = 0.884), number of hospital admissions, rates of perforation, and length of hospital stay, but routine imaging was associated with an increased proportion of patients treated for AA (53.4% versus 46.3%, *p* = 0.020) [75].

The Adult Appendicitis Score (AAS) stratifies patients into three groups: high, intermediate, and low risk of AA [76]. The score has been shown to be a reliable tool for the stratification of patients into selective imaging, which results in a low negative appendectomy rate. In a prospective study enrolling 829 adults presenting with clinical suspicion of AA, 58% of patients with histologically confirmed AA had score value at least 16 and were classified as high probability group with 93% specificity. Patients with a score below 11 were classified as low probability of AA. Only 4% of patients with AA had a score below 11, and none of them had complicated AA. In contrast, 54% of non-AA patients had a score below 11. The area under the ROC curve was significantly larger with the new score 0.882 compared with AUC of Alvarado score 0.790 and AIR score 0.810.

In the validation study by Sammalkorpi et al. [77], the AAS score stratified 49% of all AA patients into a high-risk group with the specificity of 93.3%, whereas in the low-risk group the prevalence of AA was 7%. The same study group demonstrated that diagnostic imaging has limited value in patients with a low probability of AA according to the AAS.

#### Imaging

- US
- CT
- MRI

Estimating pre-image likelihood of AA is important in tailoring the diagnostic workup and using scoring systems to guide imaging can be helpful: low-risk adult patients according to the AIR/AAS/Alvarado scores could be discharged with appropriate safety netting. In patients younger than 40 years old, a high-probability score for acute appendicitis (AIR score 9–12, AAS ≥ 16) may be used to select patients in whom imaging, such is as CT is not needed before deciding for appendectomy, whereas the high-risk patients are likely to require surgery rather than diagnostic imaging [78]. For differentiation between uncomplicated and complicated appendicitis, imaging is most likely still needed. Intermediate-risk patients benefit from systematic diagnostic imaging [79].

A positive US would lead to a discussion of appendectomy and a negative test to either CT or further clinical observation with repeated US. A conditional CT strategy, where CT is performed after the negative US, is preferable, as it reduces the number of CT scans by 50% and will correctly identify as many patients with AA as an immediate CT strategy.

#### Imaging findings

- Diameter of the appendix > 6 mm
- Single wall thickness ≥ 3 mm
- Increased echogenicity of local mesenteric fat
- Appendicolith: hyperechoic with posterior shadowing
- Free fluid surrounding appendix
- Local abscess formation
- Enlarged local mesenteric lymph nodes
- Thickening of the peritoneum

CT is the most accurate imaging study for evaluating suspected AA and alternative etiologies of right lower quadrant pain.

Recent studies from the Finnish group led by Sippola et al. [80] demonstrated that the diagnostic accuracy of contrast-enhanced low-dose CT is not inferior to standard CT in diagnosing AA or distinguishing between uncomplicated and complicated AA, enabling significant radiation dose reduction.

It should also be remembered that in many parts of the world mainly in LMICs, non-enhanced CT becomes the only option and that according to the review from Xiong et al., non-enhanced CT has a high diagnostic accuracy in detecting AA, which is adequate for clinical decision-making [81].

In pregnant women, ultrasound is initially preferred, with MRI as a second imaging examination in inconclusive cases.

### Treatment

#### Uncomplicated appendicitis

- Laparoscopic appendectomy (current standard surgical treatment where appropriate resources and skills are available) or open appendectomy. Post-operative antibiotics are unnecessary if source control is adequate.
- Antibiotic therapy without surgery (in selected patients).

Although the standard treatment for acute appendicitis has historically been appendectomy and it is the goal standard, the medical community has recently seen a notable increase in the use of antibiotic therapy as a primary means of treatment. Antibiotic therapy is a safe means of primary treatment for patients with uncomplicated acute appendicitis, without an appendicolith, but it is less effective in the long-term due to significant recurrence rates, requires a CT proven diagnosis of uncomplicated appendicitis [82, 83], and there may be a risk of perforation increasing with preoperative delay. Antibiotic therapy without surgery should be reserved for selected patients.

#### Complicated appendicitis

- Laparoscopic appendectomy (current standard surgical treatment where appropriate resources and skills are available) or open appendectomy, and antibiotic therapy for 4 days if source control is adequate (immunocompetent patients).

The advent of laparoscopy has changed the surgical treatment of AA in high-income countries. In contrast, in many areas of the world, the challenges posed by the burden of primary healthcare concerns have limited support for the development of modern tertiary healthcare facilities, and laparoscopic surgery is practiced in only a few tertiary hospitals. In the last years, several prospective randomized studies, meta-analyses, and systematic critical reviews have been published on the topic of laparoscopic appendectomy. Laparoscopic appendectomy is safe and effective, but open surgery still confers benefits, in particular with regard to the likelihood of postoperative intra-abdominal abscess. Sauerland et al. [84] have performed a meta-analysis including 67 studies, of which 56 randomized controlled trials comparing LA (with or without diagnostic laparoscopy) versus OA in adults wound infections were less likely after LA compared to OA. The incidence of intra-abdominal abscesses was increased following LA.

However, in the cumulative meta-analysis by Ukai et al. [85], with regard to the rate of intra-abdominal abscess, randomized trials published up to and including 2001 demonstrated statistical significance in favor of open appendectomy. However, the effect size in favor of open appendectomy began to disappear after 2001, demonstrating a possible inverse correlation between increased experience in laparoscopic appendectomy and decreased rate of postoperative abscesses.

In the setting of complicated appendicitis, a short course of antibiotic therapy after adequate source control is a reasonable option. The prospective trial by Sawyer et al. demonstrated that in patients with complicated IAIs undergoing an adequate source control, the outcomes after approximately 4 days of fixed-duration antibiotic therapy were similar to those after a longer course of antibiotics that extended until after the resolution of physiological abnormalities [37].

Patients who have ongoing signs of infection or systemic illness beyond 5 to 7 days of antibiotic treatment warrant a diagnostic investigation.

In Table 7, the clinical pathway for patients with acute appendicitis is illustrated.

**Table 7:** Clinical pathway for patients with acute appendicitis

Acute appendicitis	
Clinical signs and symptoms	
• Abdominal pain: it usually has a gradual onset and increases intensity over time. It is usually relived in the supine position and aggravated by coughing or abdominal movements. Typically, there may be a short history (1 to 3 days) of migration of the pain from the peri-umbilical region to the right iliac fossa	Diagnosis
• Nausea and/or vomiting soon after abdominal pain begins	
• Fever	
• Tenderness localized in the RLQ (often in complicated acute appendicitis)	
Laboratory markers	
• Increased white blood cell count	
• Leucocyte shift to left (> 75%)	
• Increased C-reactive protein) useful in predicting the risk of complicated acute appendicitis	
Scores	
• Alvarado score	
• Andersson appendicitis inflammatory response (AIR)	
Adult appendicitis score (AAS)	
• Imaging	
• US	
• CT	
• MRI	
Uncomplicated appendicitis	Treatment
• Laparoscopic appendectomy (current standard surgical treatment where appropriate resources and skills are available) or open appendectomy. Post-operative antibiotics are unnecessary if source control is adequate.	
• Antibiotic therapy without surgery (in selected patients).	
Complicated appendicitis	
• Laparoscopic appendectomy (current standard surgical treatment where appropriate resources and skills are available) or open appendectomy, and antibiotic therapy for 4 days if source control is adequate.	
Amoxicillin/clavulanate 2.2 g every 8 h +/− gentamicin 5–7 mg/Kg every 24 h	Antibiotic therapy
*Avoid Amoxicillin/clavulanate if local Enterobacteriaceae resistances > 20%*	
Piperacillin/tazobactam 6 g/0.75 g LD then 4 g/0.5 g every 6 h or 16 g/2 g by continuous infusion +/− gentamicin 5–7 mg/Kg every 24 h (in critically ill patients)	
Cefuroxime 1.5 g every 8 h + metronidazole 500 every 8 h	
Ceftriaxone 2 g every 24 h + metronidazole 500 mg every 8 h	
Cefotaxime 2 g every 8 h + metronidazole 500 mg every 8 h	
or	
In patients with beta-lactam allergy	
A fluoroquinolone-based regimen	
Ciprofloxacin 400 mg every 8/12 h + metronidazole 500 mg every 8 h	
or	
An aminoglycoside-based regimen	
Amikacin 15–20 mg/kg every 24 h + metronidazole 500 mg every 8 h	
or	
In patients at high risk for infection with community-acquired ESBL-producing Enterobacteriaceae	
Ertapenem 1 g every 24 h	

#### Antibiotic treatment

*Empiric antibiotic regimens. Normal renal function*

One of the following intravenous antibiotics

Amoxicillin/clavulanate 2.2 g every 6–8 h +/− gentamicin 5–7 mg/Kg every 24 h

*Increasing rates of antimicrobial resistance to amoxicillin/clavulanate among E. coli and other Enterobacteriaceae worldwide, during the last decade, has compromised the clinical utility of this agent for empirical therapy of serious Gram-negative infections, and therefore, it should be used based on local rates of resistance. Avoid its use if local Enterobacteriaceae resistances > 20%.*

Piperacillin/tazobactam 6 g/0.75 g LD then 4 g/0.5 g every 6 h or 16 g/2 g by continuous infusion +/− Gentamicin 5-7 mg/Kg every 24 h (in critically ill patients)

Cefuroxime 1.5 g every 8 h + metronidazole 500 every 8 h

Ceftriaxone 2 g 24-hourly + metronidazole 500 mg every 8 h

Cefotaxime 2g every 8 h + metronidazole 500 mg every 8 h

or

In patients with beta-lactam allergy

A fluoroquinolone-based regimen

Ciprofloxacin 400 mg every 8/12 h + metronidazole 500 mg every 8 h

or

An aminoglycoside-based regimen

Amikacin 15–20 mg/kg every 24 h + metronidazole 500 mg every 8 h

or

In patients at high risk for infection with community-acquired ESBL-producing Enterobacteriaceae

Ertapenem 1 g every 24 h

Tigecycline 100 mg LD, then 50 mg every 12 h (carbapenem-sparing strategy).

## Acute calculous cholecystitis

Cholelithiasis is a common disease worldwide [86, 87]. Its prevalence varies widely by region: in Western countries, the prevalence of gallstone disease ranges from approximately 7.9% in men to 16.6% in women [87]; in Asia, it ranges from approximately 3 to 15%, is nearly non-existent (less than 5%) in Africa [88], and ranges from 4.2 to 11% in China.

Acute cholecystitis develops in 1–3% of patients with symptomatic gallstones [89].

The pathogenesis of cholecystitis most commonly involves the impaction of gallstones in the bladder neck, or the cystic duct; gallstones are not always present in cholecystitis, however. Pressure on the gallbladder increases, the organ becomes enlarged, the walls thicken, the blood supply decreases, and an exudate may form. The gallbladder can become infected and can undergo necrosis and gangrene. If left untreated, it may result in perforation of the gallbladder, a rare but life-threatening phenomenon.

In 2020, the WSES guidelines for the management of acute calculous cholecystitis (AAC) were published [90].

### Diagnosis

#### Clinical signs and symptoms

- Abdominal pain in the right upper quadrant of the abdomen. The pain is characteristically worst when palpating the upper right quadrant of the abdomen during deep inspiration by the patient (Murphy’s sign)
- Fever
- Abdominal tenderness, palpable gallbladder lump (sign of complicated acute cholecystitis)

#### Laboratory markers

- Increased white blood cell count
- Leucocyte shift to left (> 75%)
- C-reactive protein

#### Imaging

- US
- CT
- MRI

#### Imaging findings

- Pericholecystic fluid (fluid around the gallbladder)
- Distended gallbladder, edematous gallbladder wall
- Gallstones (impacted in cystic duct)
- Murphy’s sign can be elicited on ultrasound examination

US is the investigation of choice in patients suspected of having acute cholecystitis.

A meta-analysis by Kiewiet et al. included studies on CT, MRI, and hepatobiliary iminodiacetic acid (HIDA) scan in addition to those on US [91]. Data on the diagnostic accuracy of CT is limited. Kiewiet et al. identified only one study including 49 patients. CT findings of acute cholecystitis included gallbladder distension (41%), gallbladder wall thickening (59%), peri-cholecystic fat density (52%), peri-cholecystic fluid collection (31%), sub-serosal edema (31%) and high gallbladder bile attenuation (24%) [92]. Thus, there is no single CT feature which is useful in the diagnosis of ACC. Furthermore, the ionizing radiation to which patients are exposed is an issue. CT is therefore usually indicated when sonography is non-diagnostic or in patients with confusing signs and symptoms [93]. Kiewiet et al. included three studies on MRI including a total of 131 patients [91]. Summary sensitivity was 85% (95% CI: 66 to 95%) and specificity was 81% (95% CI: 69 to 90%). There was substantial heterogeneity for sensitivity (I2 = 65%) and no heterogeneity for specificity (I2 = 0%). In a head-to-head comparison, the diagnostic accuracy of MRI was comparable with that of US. The comparison was however based on two studies including only 59 patients; therefore, the strength of evidence is low.

### Treatment

#### Uncomplicated cholecystitis

- Early (within 7–10 days) laparoscopic/open cholecystectomy (early treatment). Post-operative antibiotics are unnecessary if source control is adequate.
- Antibiotic therapy and planned delayed laparoscopic/open cholecystectomy (delayed treatment) (second option).

Treatment is predominantly surgical, although the timing of surgery without evidence of gangrene or perforation has been under debate in recent years. Two approaches are available for the treatment of acute cholecystitis: the early option, generally within 7 days of onset of symptoms, includes laparoscopic/open cholecystectomy to provide immediate, definitive surgical treatment after establishing the diagnosis and surgical fitness of the patient in the same hospital admission while the delayed treatment option is performed in a second hospital admission after an interval of 6–12 weeks during which time the acute inflammation settles [94, 95].

Multiple prospective trials have demonstrated that laparoscopic cholecystectomy is a safe and effective treatment for acute cholecystitis [96–99]. In addition, there is a strong population evidence base that same-admission cholecystectomy rather than delayed surgery is less costly and more effective for the health service.

As a result, immediate laparoscopic cholecystectomy has largely become the therapy of choice for acute cholecystitis in patients who are good surgical candidates.

In a recent systematic review and meta-analysis by Lyu et al. [100] including 15 RCTs and 1669 patients, early laparoscopic cholecystectomy appeared as safe and effective as delayed cholecystectomy for acute cholecystitis within 7 days from the presentation. No significant differences between the two treatment strategies were found in terms of bile duct injuries, wound infection, total complications, or conversion to open surgery. However, the pooled results showed that early surgical treatment was associated with a significantly shorter duration of hospital stay, but with no significant difference in postoperative hospital stay

While the laparoscopic approach is common, several risk factors predicting the need to convert to an open approach are reported. In the large CholeS study of 8820 patients, a prediction score was developed of six significant predictors: age (*p* = 0.005), sex (*p* < 0.001), indication for surgery (*p* < 0.001), ASA (*p* < 0.001), thick-walled gallbladder (*p* = 0.040), and CBD diameter (*p* = 0.004) which allows surgeons to predict a sixfold increase in the need for open surgery [101].

In addition, evidence from a meta-analysis summarizing 11 RCTs containing 14,645 patients, reported age > 65 years, male gender, acute cholecystitis, thickened gallbladder wall, diabetes mellitus, and previous upper abdominal surgery all as significant risks, associated with increased risk of conversion [102]. However, open cholecystectomy remains a feasible option, particularly in low-income countries [103], or elsewhere in the setting of resource limitations. The CIAOW study showed that open cholecystectomy among patients with complicated cholecystitis was the most frequently performed procedure [104]. However, laparoscopic techniques to avoid the need for conversion to open surgery include the use of subtotal cholecystectomy, gallbladder drainage, and other “bail out” strategies [105].

#### Complicated cholecystitis

- Laparoscopic cholecystectomy, with open cholecystectomy as an alternative, and antibiotic therapy for 4 days
- Cholecystostomy may be an option for acute cholecystitis in critically ill with multiple comorbidities and unfit for surgery patients who do not show clinical improvement after antibiotic therapy for 3–5 days. However current evidence shows that cholecystostomy is inferior to cholecystectomy in terms of major complications for patients with an APACHE score of 7–14.

Acute cholecystitis in elderly and critically ill patients remains a real challenge to treat. Despite the low rate of surgical impact from the laparoscopic approach, many patients are unfit for any surgery. In this subgroup of patients, urgent cholecystostomy (percutaneous transhepatic gallbladder drainage) with or without delayed laparoscopic cholecystectomy appears to be the correct clinical approach [106].

Cholecystostomy should be used for critically ill patients who are not fit for surgery, or for those who fail to improve after 2–3 days of antibiotic treatment [107]. In Table 8, the clinical pathway for patients with acute calculous cholecystitis is illustrated.

**Table 8:** Clinical pathway for patients with acute calculous cholecystitis

Acute calculous cholecystitis	
• Clinical signs and symptoms	Diagnosis
• Abdominal pain in the right upper quadrant of the abdomen. The pain is characteristically worst when palpating the upper right quadrant of the abdomen during deep inspiration by the patient (Murphy’s sign)	
• Fever	
• Absence of vomiting	
• Abdominal tenderness (sign of complicated acute cholecystitis)	
Laboratory markers	
• Increased white blood cell count	
• Leucocyte shift to left (> 75%)	
• C-reactive protein	
Imaging	
• US	
• CT	
• MRI	
Uncomplicated Colecystitis	Treatment
• Early (within 7-10 days) laparoscopic/open cholecystectomy (Early treatment). Post-operative antibiotics are unnecessary if source control is adequate.	
• Antibiotic therapy and planned delayed laparoscopic/open cholecystectomy (delayed treatment).	
Complicated cholecystitis	
• Laparoscopic cholecystectomy, with open cholecystectomy as an alternative, and antibiotic therapy for 4 days	
• Cholecystostomy may be an option for acute cholecystitis in critically ill with multiple comorbidities and unfit for surgery patients who do not show clinical improvement after antibiotic therapy for 3-5 days. However current evidence shows that cholecystostomy is inferior to cholecystectomy in terms of major complications for patients with an APACHE score of 7–14 in terms of major complications.	
Amoxicillin/clavulanate 2.2 g every 8 h +/− gentamicin 5–7 mg/Kg every 24 h	Antibiotic therapy
*Avoid Amoxicillin/clavulanate if local Enterobacteriaceae resistances > 20%.* Piperacillin/tazobactam 6 g/0.75 g LD then 4 g/0.5 g every 6 h or 16 g/2 g by continuous infusion +/− gentamicin 5–7 mg/Kg every 24 h (in critically ill patients)	
Ceftriaxone 2 g every 24 h + metronidazole 500 mg every 8 h	
Cefotaxime 2g every 8 h + metronidazole 500 mg every 8 h	
or	
In patients with beta-lactam allergy	
A fluoroquinolone-based regimen	
Ciprofloxacin 400 mg every 8/12 h + metronidazole 500 mg every 8 h	
or	
An aminoglycoside-based regimen	
Amikacin 15-20 mg/kg every 24 h + metronidazole 500 mg every 8 h	
or	
In patients at high risk for infection with community-acquired ESBL-producing	
Enterobacteriaceae	
One of the following antibiotics	
Tigecycline 100 mg LD, then 50 mg every 12 h (carbapenem-sparing strategy)	
Ertapenem 1 g every 24 h	
Meropenem 1 g every 8 h (only in patients with septic shock)	
Doripenem 500 mg every 8 h (only in patients with septic shock)	
Imipenem/cilastatin 500 mg every 6 h (only in patients with septic shock)	
In patients at high risk for infection from enterococci including immunocompromised patients or patients with recent antibiotic exposure, consider the use of ampicillin 2 g every 6 h if patients are not being treated with piperacillin/tazobactam or imipenem/cilastatin (active against ampicillin-susceptible enterococci) or tigecycline	

#### Antibiotic treatment

*Empiric antibiotic regimens. Normal renal function*

One of the following intravenous antibiotics

Amoxicillin/clavulanate 2.2 g every 8 h

*Increasing rates of antimicrobial resistance to amoxicillin/clavulanate among E. coli and other Enterobacteriaceae worldwide, during the last decade, has compromised clinical utility of this agent for empirical therapy of serious Gram-negative infections and therefore it should be used based on local rates of resistance. Avoid its use if Enterobacteriaceae resistances > 20%.*

Piperacillin/tazobactam 6 g/0.75 g LD then 4 g/0.5 g every 6 h or 16 g/ 2 g by continuous infusion, (in critically ill patients)

Ceftriaxone 2 g every 24 h + metronidazole 500 mg every 8 h

Cefotaxime 2g every 8 h + metronidazole 500 mg every 8 h

or

In patients with beta-lactam allergy

A fluoroquinolone-based regimen

Ciprofloxacin 400 mg every 8 or 12 h + metronidazole 500 mg every 8 h

or

An aminoglycoside-based regimen

Amikacin 15–20 mg/kg every 24 h + metronidazole 500 mg every 8 h

or

In patients at high risk for infection with community-acquired ESBL-producing Enterobacteriaceae

One of the following antibiotics

Tigecycline 100 mg LD, then 50 mg every 12 h (carbapenem-sparing strategy)

Ertapenem 1 g every 24 h

Meropenem 1 g every 8 h (only in patients with septic shock)

Doripenem 500 mg every 8 h (only in patients with septic shock)

Imipenem/cilastatin 500 mg every 6 h (only in patients with septic shock)

In patients at high risk for infection from enterococci including immunocompromised patients or patients with recent antibiotic exposure, consider the use of ampicillin 2 g every 6 h if patients are not being treated with piperacillin/tazobactam or imipenem/cilastatin (active against ampicillin-susceptible enterococci) or tigecycline.

## Acute cholangitis

Acute cholangitis is an infectious disease characterized by acute inflammation and infection in the bile ducts resulting from a combination of biliary obstruction and bacterial growth in bile.

Bacteria reach the biliary system either by ascent from the intestine or by the portal venous system [108]. The most common cause of cholangitis is choledocholithiasis [109].

### Diagnosis

#### Clinical signs and symptoms

- Intermittent fever with rigors
- Jaundice
- Right upper quadrant abdominal pain

#### Laboratory markers

- Increased white blood cell count
- C-reactive protein
- Total bilirubin
- Direct bilirubin
- Alkaline phosphatase
- Gamma-glutamyl transpeptidase
- Aspartate aminotransferase
- Alanine aminotransferase

#### Imaging

- US
- CT
- MRI
- Endoscopic ultrasound (EUS)
- ERCP

#### Imaging findings

- Dilatation of intra- and extrahepatic bile ducts
- Thickening of the bile duct wall
- Intraluminal stones or sludge

Historically, US has been the initial modality for evaluating biliary obstruction in the setting of suspected cholangitis. However, research and recommendations published over the last decade have challenged this approach, and data have become increasingly supportive of the use of computed tomography (CT) as the initial imaging study of choice to confirm biliary obstruction and identify its source. In case the cause of biliary obstruction presenting with cholangitis is not identified on US and CT, EUS is very helpful in diagnosing occult choledocholithiasis. EUS is more sensitive than both CT and MRI in these settings. ERCP has similar sensitivity to EUS and it has therapeutic potential. Documentation of pus during ERCP is the gold standard for diagnosis of cholangitis but ERCP can worsen cholangitis if there is inadvertent contrast injection into an undrained segment.

### Treatment

- Biliary drainage and antibiotic therapy for 3–5 days

The key elements of therapy in acute cholangitis are adequate antimicrobial treatment to avoid or manage the septic complications and biliary decompression to restore biliary drainage in case of obstruction [110]. The clinical presentation varies, and initial risk stratification is important to guide further management [111].

In severe cholangitis, an early interventional approach is absolutely essential for survival.

The type and timing of biliary drainage should be based on the severity of the clinical presentation, and the availability and feasibility of drainage techniques, such as endoscopic retrograde cholangiopancreatography (ERCP), percutaneous transhepatic cholangiography (PTC), and open surgical drainage.

- Endoscopic retrograde cholangiopancreatography (ERCP) plays a central role in the management of biliary obstruction in patients with acute cholangitis and is the treatment of choice for biliary decompression in patients with moderate/severe acute cholangitis. Biliary endoscopic sphincterotomy with stone extraction continues to be a therapeutic choice and the reference standard in the treatment of symptomatic choledocholithiasis, especially for the solitary common bile duct stones up to 10–12 mm in diameter. It has been shown that biliary endoscopic sphincterotomy reduces hospital stay and duration of cholangitis but does not prevent recurrent cholangitis. Biliary sphincterotomy in acute cholangitis as a result of common bile duct stones can allow definitive therapy or placement of a large-bore biliary stent which can drain the thick infected bile more efficiently. Endoscopic drainage can be carried out either by external drainage using a nasobiliary tube or internal drainage using biliary stenting. Both the modalities of internal and external drainage have their own advantages and disadvantages. Both have similar technical and clinical success rates.
- Percutaneous transhepatic biliary drainage (PTBD) should be reserved for patients in whom ERCP fails. PTBD can lead to significant complications, including biliary peritonitis, haemobilia, pneumothorax, hematoma, liver abscesses, and patient discomfort related to the catheter.
- Open drainage may be indicated for patients who cannot undergo non-invasive drainage procedures, for anatomical and structural reasons, including patients after Roux-en-Y choledochojejunostomy or patients with a propensity for hemorrhage. In open drainage, the goal is to decompress the biliary system. Simple procedures such as T-tube placement without choledocholithotomy should be recommended as a temporary solution in unstable patients, because prolonged operations should be avoided in such ill patients. Whenever possible, definitive elimination of the biliary obstruction should be the goal of surgery.

Endoscopic retrograde cholangiopancreatography (ERCP) is the treatment of choice for biliary decompression in patients with moderate/severe acute cholangitis.

A randomized controlled trial (RCT) [112] was conducted to compare endoscopic and open drainage in 82 patients with severe acute cholangitis with hypotension and disturbed consciousness. This RCT demonstrated that the morbidity and mortality of endoscopic naso-biliary drainage (ENBD) + endoscopic sphincterotomy (EST; *n* = 41) were significantly lower than those of T-tube drainage under laparotomy (*n* = 41).

There are various endoscopic transpapillary options available, including biliary stent or nasobiliary drain placement above the obstruction site ± sphincterotomy, all of which with appropriate indications corresponding to disease severity and clinical context [113].

Endoscopic biliary decompression by nasobiliary catheter or indwelling stent was equally effective for patients with acute suppurative cholangitis caused by bile duct stones in a prospective randomized trial published in 2002 [114]. The indwelling stent was associated with less post-procedure discomfort and avoided the potential problem of inadvertent removal of the nasobiliary catheter.

Percutaneous transhepatic biliary drainage (PTBD) should be reserved for patients in whom ERCP fails either due to cannulation, or an inaccessible papilla.

However, PTBD can lead to significant complications, including biliary peritonitis, hemobilia, pneumothorax, hematoma, liver abscesses, and patient discomfort related to the catheter [115].

In 2012, a retrospective study comparing the safety and effectiveness of endoscopic and percutaneous transhepatic biliary drainage in the treatment of acute obstructive suppurative cholangitis was reported. It confirmed the clinical efficacy of endoscopic drainage as well as its ability to facilitate subsequent endoscopic or surgical intervention [116]. Surgical biliary drainage should only be used in patients for whom endoscopic or percutaneous trans-hepatic drainage is contraindicated or those in whom it has been unsuccessfully performed.

#### Antibiotic treatment

Although there are no clinical data to support the use of antibiotics with biliary penetration for these patients, the efficacy of antibiotics in the treatment of biliary infections may also depend on effective biliary antibiotic concentrations [117]. Obviously, in patients with obstructed bile ducts, the biliary penetration of antibiotics may be poor and effective biliary concentrations are reached only in a minority of patients [86].

Antibiotics commonly used to treat biliary tract infections and their biliary penetration ability are illustrated in Table 9.

**Table 9:** Biliary penetration ability of the most common antibiotics

Good penetration efficiency	Low penetration efficiency
Piperacillin/tazobactam	Ceftriaxone
Tigecycline	Cefotaxime
Amoxicillin/clavulanate	Meropenem
Ciprofloxacin	Ceftazidime
Ampicillin/sulbactam	Vancomycin
Cefepime	Amikacin
Levofloxacin	Gentamicin

In Table 10, the clinical pathway for patients with acute calculous cholecystitis is illustrated.

**Table 10:** Clinical pathway for patients with acute cholangitis is illustrated

Acute cholangitis	
Clinical signs and symptoms	Diagnosis
• Intermittent fever with rigors	
• Jaundice	
• Right upper quadrant abdominal pain	
Laboratory markers	
• Increased white blood cell count	
• C-reactive protein	
• Total bilirubin and direct bilirubin	
• Alkaline phosphatase	
• Gamma-glutamyl transpeptidase	
• Aspartate aminotransferase and Alanine aminotransferase	
Imaging	
• US	
• CT	
• MRI	
• Endoscopic ultrasound (EUS)	
• ERCP	
• Endoscopic retrograde cholangiopancreatography (ERCP). Endoscopic drainage can be carried out either by external drainage using a nasobiliary tube or internal drainage using biliary stenting. Both the modalities of internal and external drainage have their own advantages and disadvantages. Both have similar technical and clinical success rates.	Treatment
• Percutaneous transhepatic biliary drainage (PTBD) should be reserved for patients in whom ERCP fails. PTBD can lead to significant complications, including biliary peritonitis, haemobilia, pneumothorax, hematoma, liver abscesses, and patient discomfort related to the catheter.	
• Open drainage may be indicated for patients who cannot undergo such noninvasive drainage procedures, for anatomical and structural reasons, including patients after Roux-en-Y choledochojejunostomy with a propensity for hemorrhage.	
Amoxicillin/clavulanate 2.2 g every 6/8 h +/− gentamicin 5–7 mg/Kg every 24 h	Antibiotic therapy
*Avoid Amoxicillin/clavulanate if local Enterobacteriaceae resistances > 20%.*	
Piperacillin/tazobactam 6 g/0.75 g LD then 4 g/0.5 g every 6 h or 16 g/2 g by	
continuous infusion +/− gentamicin 5-7 mg/Kg every 24 h (in critically ill patients)	
Ceftriaxone 2 g every 24 h + metronidazole 500 mg every 8 h	
Cefotaxime 2 g every 8 h + metronidazole 500 mg every 8 h	
or	
In patients with beta-lactam allergy	
A fluoroquinolone-based regimen	
Ciprofloxacin 400 mg every 8/12 h + metronidazole 500 mg every 8 h	
or	
In patients at high risk for infection with community-acquired ESBL-producing Enterobacteriaceae	
One of the following antibiotics	
Tigecycline 100 mg LD, then 50 mg every 12 h (carbapenem-sparing strategy)	
Ertapenem 1 g every 24 h	
Meropenem 1 g every 8 h (only in patients with septic shock)	
Doripenem 500 mg every 8 h (only in patients with septic shock)	
Imipenem/cilastatin 500 mg every 6 h (only in patients with septic shock)	
In patients at high risk for infection from enterococci including immunocompromised patients or patients with recent antibiotic exposure, consider the use of ampicillin 2 g every 6 h if patients are not being treated with Piperacillin/tazobactam or imipenem/cilastatin (active against ampicillin-susceptible enterococci) or tigecycline.	

*Empiric antibiotic regimens. Normal renal function*

One of the following intravenous antibiotics

Amoxicillin/clavulanate 2.2 g every 8 h

*Increasing rates of antimicrobial resistance to amoxicillin/clavulanate among E. coli and other Enterobacteriaceae worldwide, during the last decade, has compromised clinical utility of this agent for empirical therapy of serious Gram-negative infections and therefore it should be used based on local rates of resistance. Avoid its use if Enterobacteriaceae resistances > 20%.* Piperacillin/tazobactam 6 g/0.75 g LD then 4 g/0.5 g every 6 h or 16 g/2 g by continuous infusion, (in critically ill patients)

Ceftriaxone 2 g every 24 h + metronidazole 500 mg every 8 h

Cefotaxime 2 g every 8 h + metronidazole 500 mg every 8 h

or

In patients with beta-lactam allergy

A fluoroquinolone-based regimen

Ciprofloxacin 400 mg every 8 or 12 h + metronidazole 500 mg every 8 h

or

In patients at high risk for infection with community-acquired ESBL-producing Enterobacteriaceae

One of the following intravenous antibiotics

Tigecycline 100 mg LD, then 50 mg every 12 h (carbapenem-sparing strategy)

Ertapenem 1 g every 24 h (not active against *Pseudomonas aeruginosa*)

Meropenem 1 g every 8 h (only in patients with septic shock)

Doripenem 500 mg every 8 h (only in patients with septic shock)

Imipenem/cilastatin 500 mg every 6 h (only patients with septic shock)

In patients at high risk for infection from enterococci including immunocompromised patients or patients with recent antibiotic exposure, consider the use of ampicillin 2 g every 6 h if patients are not being treated with piperacillin/tazobactam or imipenem/cilastatin (active against ampicillin-susceptible enterococci) or tigecycline.

## Acute left colonic diverticulitis

Acute left colonic diverticulitis (ALCD) is a common problem encountered by surgeons in the acute setting. It encompasses a variety of conditions, ranging from localized diverticular inflammation to perforation and fecal peritonitis. Decisions in the diagnosis and treatment of acute diverticulitis often depend on clinicians’ personal preferences rather than evidence-based medicine.

In 2020, WSES guidelines for the management of ALCD were updated [118].

### Classifications

ALCD ranges in severity from uncomplicated inflammatory diverticulitis to complicated diverticulitis (abscess formation or perforation). For the past three decades, the Hinchey classification has been the most commonly used classification for complicated ALCD in the international literature.

Based on the surgical findings of abscesses and peritonitis, Hinchey classified the severity of acute diverticulitis into four grades [119], but this classification in its original form is not used anymore.

The management of ALCD has recently changed dramatically, due to better radiological imaging and the availability of non-surgical treatment options. Computer tomography (CT) imaging has become the primary diagnostic tool in the diagnosis and staging of patients with acute diverticulitis and more detailed information provided by CT scans led to several modifications of the Hinchey classification.

In 2002, Ambrosetti et al. [120] classified diverticulitis into severe or moderate disease. In this classification, the CT scan determined the grade of severity guiding the physician in the treatment of acute complications. Moderate diverticulitis was defined by wall thickening of ≥ 5 mm and signs of pericolic fat inflammation. Severe diverticulitis was defined by wall thickening accompanied by abscess formation, extraluminal air, or extraluminal contrast extravasation.

Moderate diverticulitis

- Localized sigmoid wall thickening
- Pericolic fat stranding

Severe diverticulitis

- Abscess
- Extraluminal air
- Extraluminal contrast

In 2005, Kaiser et al. [121] modified the Hinchey classification according to specific CT findings:

- Stage 0 mild clinical diverticulitis.
- Stage 1a confined pericolic inflammation.
- Stage 1b confined pericolic abscess.
- Stage 2 pelvic or distant intra-abdominal abscess.
- Stage 3 generalized purulent peritonitis.
- Stage 4 fecal peritonitis at presentation.

In 2015, Sallinen et al. [122] published an interesting retrospective study of patients treated for diverticulitis, setting the stage for the treatment of acute diverticulitis based on clinical, radiologic, and physiologic parameters. They included 5 stages:

- Stage 1 Uncomplicated diverticulitis.
- Stage 2 Complicated diverticulitis with small abscess (<6 cm).
- Stage 3 Complicated diverticulitis with large abscess (≥6 cm) or distant intraperitoneal or retroperitoneal air.
- Stage 4 Generalized peritonitis without organ dysfunction.
- Stage 5 Generalized peritonitis with organ dysfunction.

A proposal for a CT-guided classification of left colon acute diverticulitis was published in 2015 by the WSES acute diverticulitis working group. It is a simple classification system of acute diverticulitis based on CT scan findings [118]. It may guide clinicians in the management of acute diverticulitis and may be universally accepted for day-to-day practice. The WSES classification divides acute diverticulitis into 2 groups: uncomplicated and complicated. In the event of uncomplicated acute diverticulitis, the infection does not extend to the peritoneum. In the event of complicated acute diverticulitis, the infectious process proceeds beyond the colon. Complicated acute diverticulitis is divided into 4 stages, based on the extension of the infectious process:

Uncomplicated:

- Stage 0 Diverticula, thickening of the colonic wall or increased density of the pericolic fat.

Complicated:

- Stage 1a Pericolic air bubbles or little pericolic fluid without abscess (within 5 cm from inflamed bowel segment).
- Stage 1b Abscess ≤ 4 cm.
- Stage 2a Abscess > 4 cm.
- Stage 2b Distant air (>5 cm from inflamed bowel segment).
- Stage 3 Diffuse fluid without distant free air (no hole in colon).
- Stage 4 Diffuse fluid with distant free air (persistent hole in colon).

### Diagnosis

#### Clinical signs and symptoms

- Abdominal pain in the left lower quadrant of the abdomen without vomiting
- Elevated temperature
- Tenderness localized in the left lower quadrant

#### Laboratory markers

- Increased white blood cell count
- Leucocyte shift to left (>75%)
- C-reactive protein

#### Imaging

- US
- CT

#### Imaging findings

- Intestinal wall thickening.
- Signs of inflammation in the pericolonic fat and thickening of the lateroconal fascia.
- Signs of intestinal perforation (extraluminal gas, intra-abdominal fluid).
- Pericolonic or distant abscess.

Patients who fulfill the triad of criteria CRP > 50 mg/L, absence of vomiting, and tenderness in the left lower quadrant are highly likely to have acute diverticulitis. The positive predictive value of this triad is very high in the emergency department development cohort (97%) and excellent in an independent validation cohort (100%) [123].

Radiological imaging techniques that are used for diagnosing ALCD in the emergency setting are US and CT [124]. Currently, CT is the established method of choice when compared to US and most guidelines cite the high accuracy and other advantages of CT in diagnosing complicated disease and detailed assessment of complications. This approach is the gold standard for both the diagnosis and staging of ALCD due to its excellent sensitivity and specificity [125, 134]. CT scan can also rule out other diagnoses such as ovarian pathology, or leaking aortic or iliac aneurysms.

CT findings in patients with ALCD may include diverticulosis with associated colon wall thickening, fat stranding, phlegmon, extraluminal gas, abscess formation, or intra-abdominal free fluid [126]. CT imaging can go beyond the accurate diagnosis of ALCD. CT criteria may also be used to determine the grade of severity and may drive treatment planning [120]. US is a real-time dynamic examination with wide availability and easy accessibility [127]. Its limitations include operator dependency, poor assessment in obese patients, and difficulty in the detection of free gas and deeply located abscesses [128].

A systematic review and meta-analysis of studies [203] that reported diagnostic accuracy of the clinical diagnosis and diagnostic modalities in patients with suspected diverticulitis was published in 2014. Summary sensitivity estimates for US were 90% (95% CI 76–98%) versus 95% (95% CI 91–97%) for CT (*p* = 0.86). Summary specificity estimates for US were 90% (95% CI 86–94%) versus 96% (95% CI 90–100%) for CT (*p* = 0.04).

Although CT is the most sensitive imaging modality for patients with suspected acute diverticulitis, a step-up approach with CT performed after an inconclusive or negative US has been proposed as a safe and alternative approach for patients with suspected acute diverticulitis [129, 203].

### Treatment

#### Uncomplicated diverticulitis

- Conservative treatment without antibiotics in patients with CT diagnosis of uncomplicated acute diverticulitis.
- Antibiotic therapy for 5–7 days in patients with CT diagnosis of uncomplicated acute diverticulitis is reserved for immunocompromised patients and patients with signs of sepsis.

The definition of uncomplicated acute diverticulitis is often vague and poorly defined. Uncomplicated acute diverticulitis is defined as localized diverticular inflammation without any abscess or perforation. A universally accepted classification divides IAIs into complicated and uncomplicated [9]. In uncomplicated IAIs, the infection only involves a single organ and does not extend to the peritoneum, while in complicated IAIs, the infectious process extends beyond the organ, causing either localized or diffuse peritonitis [40]. For a better definition of acute diverticulitis in these guidelines, we use the term complicated and uncomplicated according to the classification of IAIs.

Uncomplicated acute diverticulitis is an anatomically confined inflammatory process. CT findings include diverticula, thickening of the wall, and increased density of the pericolic fat. Patients with uncomplicated diverticulitis usually have an indolent course with a low incidence of subsequent complications.

The utility of antibiotics in acute uncomplicated acute diverticulitis has been a point of controversy. In recent years, several studies demonstrated that antimicrobial treatment was not superior to withholding antibiotic therapy, in terms of clinical resolution, in patients with mild unperforated diverticulitis [130]. The current consensus is that uncomplicated acute diverticulitis may be a self-limiting condition in which local host defenses can manage the inflammation without antibiotics in immunocompetent patients. In this context, antibiotics are not necessary in the treatment of uncomplicated disease.

A multicenter randomized trial was published in 2012 by Chabok et al. involving ten surgical departments in Sweden and one in Iceland recruiting 623 patients with computed tomography-confirmed acute uncomplicated left-sided diverticulitis [131]. Patients were randomized to treatment with (314 patients) or without (309 patients) antibiotics. Antibiotic treatment for acute uncomplicated diverticulitis neither accelerated recovery nor prevented complications or recurrence. Therefore, antibiotics should be reserved for the treatment of complicated diverticulitis.

A recent Dutch randomized controlled trial of observational versus systemic antibiotic treatment (DIABOLO trial) [132] for a first episode of CT-proven ALCD Hinchey stages 1a and 1b confirmed that observational treatment without antibiotics did not prolong recovery and could be considered appropriate in these patients.

Long-term follow-up of the AVOD and DIABOLO trials on omitting antibiotic treatment in uncomplicated diverticulitis have confirmed that routine antibiotics are not beneficial. A recently published individual patient data meta-analysis (IPDMA) of both RCTs shows that observational management of acute uncomplicated diverticulitis is safe [133]. Some statistical uncertainty remains, but no subgroup that would benefit from antibiotic treatment is apparent [133].

Outpatient management is suggested for patients with uncomplicated acute diverticulitis, with no comorbidities. These patients should be clinically monitored as outpatients and re-evaluated within 7 days to assess for resolution of the inflammatory processes. Earlier revaluation is necessary if the clinical condition deteriorates.

The DIVER trial [134] has demonstrated that outpatient treatment may be safe and effective in selected patients with uncomplicated acute diverticulitis and can reduce costs without negatively influencing the quality of life. The multicenter, RCT included patients older than 18 years with acute uncomplicated diverticulitis. All patients underwent abdominal CT. The first dose of antibiotic was given intravenously to all patients in the emergency department, and then, patients were either admitted to the hospital or discharged. Among a total of 132 patients, four patients in those admitted to hospital and three patients in those discharged to home management developed treatment failure (there were no differences between the groups (*p* = 0.62). The overall health care cost per episode was 3 times less in the outpatient treated group, with significant costs savings of €1124.70 per patient. No differences were observed between the groups in terms of quality of life.

A systematic review including 21 studies (11 prospective, 9 retrospective, and only 1 randomized trial) with 1781 patients with ALCD managed in the outpatient setting was recently published [135]. The meta-analysis concluded that outpatient management is safe, and the overall failure rate in an outpatient setting was 4.3% (95% CI 2.6–6.3%).

#### Abdominal abscess

- Antibiotic therapy alone in patients with small diverticular abscesses.
- Percutaneous drainage combined with antibiotic therapy for 3–5 days in large diverticular abscesses. Whenever percutaneous drainage of the abscess is not feasible or not available, based on the clinical conditions, unless emergency surgery is needed, antibiotics could be considered the primary treatment.

The size of 4–5 cm may be a reasonable limit between antibiotic treatment alone, versus percutaneous drainage combined with antibiotic treatment in the management of diverticular abscesses [136–139].

Whenever percutaneous drainage of the abscess is not feasible or not available, based on the clinical conditions, patients with large abscesses can be initially treated with antibiotic therapy alone. However, careful clinical monitoring is mandatory.

#### Diffuse peritonitis

- Primary resection and anastomosis with or without a diverting stoma (in clinically stable patients with no co-morbidities)
- Hartmann’s procedure (HP) (in critically ill patients and/or in patients with multiple major comorbidities).
- Laparoscopic peritoneal lavage and drainage suitable only for patients with purulent (but not fecal) peritonitis due to complicated diverticulitis. Very controversial.

+

Antibiotic therapy for 4 days (immunocompetent and stable patients) (more days if there are signs of ongoing infection).

HP has been considered the procedure of choice in patients with generalized peritonitis and remains a safe technique for emergency colectomy in diverticular peritonitis. It is especially useful in critically ill patients and in patients with multiple comorbidities. However, restoration of bowel continuity after a HP is associated with significant morbidity and resource utilization [140]. As a result, many of these patients do not undergo reversal surgery and remain with a permanent stoma [141].

In recent years, some authors have reported the role of primary resection and anastomosis with or without a diverting stoma, in the treatment of acute diverticulitis, even in the presence of diffuse peritonitis [142]. The decision regarding the surgical choice in patients with diffuse peritonitis is generally left to the judgment of the surgeon, who takes into account the clinical condition and the comorbidities of the patient. Studies comparing mortality and morbidity of the HP versus primary anastomosis did not show any significant differences. However, most studies had relevant selection biases, as demonstrated by some systematic reviews [143–146].

The results of a multicenter, randomized, open-label, superiority trial which was done in eight academic hospitals and 34 teaching hospitals in Belgium, Italy, and the Netherlands were published in 2019 [147]. Patients aged between 18 and 85 years who presented with clinical signs of general peritonitis and suspected perforated diverticulitis were eligible for inclusion if plain abdominal radiography or CT scan showed diffuse free air or fluid. Between July 1, 2010, and Feb 22, 2013, and June 9, 2013, and trial termination on June 3, 2016, 133 patients (93 with Hinchey III disease and 40 with Hinchey IV disease) were randomly assigned to Hartmann’s procedure (68 patients) or primary anastomosis (65 patients). 12-month stoma-free survival was significantly better for patients undergoing primary anastomosis compared with Hartmann’s procedure (94·6% [95% CI 88.7–100] versus 71.7% [95% CI 60.1–83.3], hazard ratio 2.79 [95% CI 1·86–4.18]; log-rank *p* < 0.0001). There were no significant differences in short-term morbidity and mortality after the index procedure for Hartmann’s procedure compared with primary anastomosis (morbidity: 29 [44%] of 66 patients versus 25 [39%] of 64, *p* = 0.60; mortality: two [3%] versus four [6%], *p* = 0·44).

A minimally invasive approach using laparoscopic peritoneal lavage and drainage has been proposed in recent years as an alternative to colonic resection. It can potentially avoid a stoma in patients with diffuse peritonitis. It consists of the laparoscopic aspiration of pus followed by abdominal lavage and the placement of abdominal drains, which remain for many days after the procedure.

Laparoscopic lavage reduces the risk for colostomy at one- and two-year follow-up, but may in the short-term result in intraabdominal abscesses and overlooked free or tumor perforations requiring reoperation. Laparoscopic lavage consists of the laparoscopic aspiration of pus followed by abdominal lavage and optional the placement of abdominal drains that, which remain for many days after the procedure.

Great debate is still open on this topic, mainly due to the discrepancy and sometimes disappointing results of the latest prospective trials such as SCANDIV, Ladies, and DILALA trials [148–152], but lower stoma rates have been noted after laparoscopic lavage for purulent peritonitis. Laparoscopic peritoneal lavage and drainage is feasible in selected patients with purulent peritonitis.

In Table 11, the clinical pathway for patients with acute diverticulitis is illustrated.

**Table 11:** Clinical pathway for patients with acute diverticulitis is illustrated

Acute diverticulitis	
Clinical signs and symptoms	Diagnosis
• Abdominal pain in the left lower quadrant of the abdomen without vomiting	
• Elevated temperature	
• Tenderness localized in the left lower quadrant	
Laboratory markers	
• Increased white blood cell count	
• Leucocyte shift to left (> 75%)	
• C-reactive protein	
Imaging	
• US	
• CT	
Uncomplicated acute diverticulitis	Treatment
• Conservative treatment without antibiotics in patients with CT diagnosis of uncomplicated acute diverticulitis.	
• Antibiotic therapy for 5–7 days in patients with CT diagnosis of uncomplicated acute diverticulitis is reserved for immunocompromised patients and patients with signs of sepsis	
Abdominal abscess	
• Antibiotic therapy alone in patients with small diverticular abscesses. Percutaneous drainage combined with antibiotic therapy for 3–5 days in large diverticular abscesses.	
• Whenever percutaneous drainage of the abscess is not feasible or not available, based on the clinical conditions, unless emergency surgery is needed, antibiotics could be considered the primary treatment.	
Diffuse peritonitis	
• Primary resection and anastomosis with or without a diverting stoma (in clinically stable patients with no co-morbidities)	
• Hartmann’s procedure (HP) (in critically ill patients and/or in patients with multiple major comorbidities).	
• Laparoscopic peritoneal lavage and drainage in patients with purulent (but not fecal) peritonitis due to complicated diverticulitis. Very controversial.	
+	
Antibiotic therapy for 4 days	
• A damage control surgical strategy may be useful for patients in physiological extremis from abdominal sepsis	
Amoxicillin/clavulanate 2.2 g every 8 h +/− gentamicin 5–7 mg/Kg every 24 h	Antibiotic therapy
*Avoid Amoxicillin/clavulanate if local Enterobacteriaceae resistances > 20%.*	
Piperacillin/tazobactam 6 g/0.75 g LD then 4 g/0.5 g every 6 h or 16 g/2 g by	
continuous infusion +/− gentamicin 5–7 mg/Kg every 24 h (in critically ill patients)	
Ceftriaxone 2 g every 24 h + metronidazole 500 mg every 8 h	
Cefotaxime 2 g every 8 h + metronidazole 500 mg every 8 h	
or	
In patients with beta-lactam allergy	
A fluoroquinolone-based regimen	
Ciprofloxacin 400 mg every 8/12 h + metronidazole 500 mg every 8 h	
or	
An aminoglycoside-based regimen	
Amikacin 15–20 mg/kg every 24 h + metronidazole 500 mg every 8 h	
or	
In patients at high risk for infection with community-acquired ESBL-producing	
Enterobacteriaceae	
One of the following antibiotics	
Tigecycline 100 mg LD, then 50 mg every 12 h (carbapenem-sparing strategy)	
Ertapenem 1 g every 24 h Meropenem 1 g every 8 h (only in patients with septic shock)	
Doripenem 500 mg every 8 h (only in patients with septic shock)	
Imipenem/cilastatin 500 mg every 6 h (only in patients with septic shock)	
In patients at high risk for infection from enterococci including immunocompromised patients or patients with recent antibiotic exposure, consider the use of ampicillin 2 g every 6 h if patients are not being treated with Piperacillin/tazobactam or imipenem/cilastatin (active against ampicillin-susceptible enterococci) or tigecycline.	

#### Antibiotic treatment for complicated diverticulitis

*Empiric antibiotic regimens. Normal renal function*

One of the following intravenous antibiotics

Amoxicillin/clavulanate 2.2 g every 8 h +/− gentamicin

*Increasing rates of antimicrobial resistance to amoxicillin/clavulanate among E. coli and other Enterobacteriaceae worldwide, during the last decade, has compromised clinical utility of this agent for empirical therapy of serious Gram-negative infections and therefore it should be used based on local rates of resistance. Avoid its use if Enterobacteriaceae resistances > 20%.*

Piperacillin/tazobactam 6 g/0.75 g LD then 4 g/0.5 g every 6 h or 16 g/2 g by continuous infusion, (in critically ill patients)

Ceftriaxone 2 g every 24 h + metronidazole 500 mg every 8 h

Cefotaxime 2 g every 8 h + metronidazole 500 mg every 8 h

or

In patients with beta-lactam allergy

A fluoroquinolone-based regimen

Ciprofloxacin 400 mg every 8 or 12 h + metronidazole 500 mg every 8 h

or

An aminoglycoside-based regimen

Amikacin 15–20 mg/kg every 24 h + metronidazole 500 mg every 8 h

or

In patients at high risk for infection with community-acquired ESBL-producing Enterobacteriaceae

One of the following intravenous antibiotics

Tigecycline 100 mg LD, then 50 mg every 12 h (carbapenem-sparing strategy)

Ertapenem 1 g every 24 h

Meropenem 1 g every 8 h (only in patients with septic shock)

Doripenem 500 mg every 8 h (only in patients with septic shock)

Imipenem/cilastatin 500 mg every 6 h (only in patients with septic shock)

Empiric antibiotic regimens. Normal renal function

In patients at high risk for infection from Enterococci including immunocompromised patients or patients with recent antibiotic exposure, consider the use of ampicillin 2 g every 6 h if patients are not being treated with piperacillin/tazobactam or imipenem/cilastatin (active against ampicillin-susceptible enterococci) or tigecycline.

#### Damage control surgery

Source control by resection of the affected segment of the colon, usually the sigmoid, followed by primary fascial closure is standard treatment. If primary fascial closure is not possible due to visceral edema, a negative pressure-based temporary closure device should be used for progressive closure steps over the next days. A damage control surgical strategy may be useful for patients in physiological extremis from abdominal sepsis [153]. The initial surgery focuses on control of the sepsis, and a subsequent operation deals with the anatomical restoration of the gastrointestinal tract, after a period of physiological resuscitation. This strategy facilitates both the control of the severe sepsis as well as potentially improving the rate of primary anastomosis [154–156]. Data on this approach in complicated diverticulitis is limited.

## Acute right colonic diverticulitis

Acute colonic diverticulitis is a common condition affecting the adult population. Traditionally, the sigmoid colon is considered the most commonly involved part, and ARCD is much rarer [157]. However, in some regions of the world, ARCD outnumbers ALCD [139]. ARCD differs from ALCD in some aspects. The former is usually solitary [158], and has a low rate of complicated diverticulitis [159].

ARCD generally occurs in middle-aged men, and its incidence does not increase with age. ARCD located in the cecum is difficult to distinguish from acute appendicitis because of their similar symptoms and signs. CT appears to be the best overall imaging modality in the diagnosis of possible ARCD [160, 161]. However, US is cheaper than CT and poses no radiation, which may be particularly important since patients having right-sided diverticulitis are relatively younger.

US features, including diverticular wall thickening, surrounding echogenic fat, and intra-diverticular echogenic material, can provide clear information for making the correct preoperative diagnosis. However, US is operator dependent. Ambiguous US studies should be complemented with a contrast-enhanced CT [162].

Currently, the management of ARCD is not well defined, and no guidelines have been proposed.

Although previous studies have shown that the percentage of complications requiring surgery is higher in patients with ALCD than in patients with ARCD [163], the principles of diagnosis and treatment of ARCD are very similar to those of ALCD. As a treatment option, non-operative methods should be preferred in cases without diffuse peritonitis, although differentiating benign and malignant cases pre-operatively is often difficult [164]. Surgical treatment is usually used in the management of complicated cases [165, 166]. Resection of the inflamed colon with primary anastomosis may be performed laparoscopically in experienced centers [167].

## Small bowel perforation

Compared to LMIC, small bowel perforations are a less common source of peritonitis in Western countries, where most small bowel perforations are due to unrecognized intestinal ischemia (mesenteric or strangulation) or inflammatory bowel disease such as Crohn’s disease. This pattern of disease is quite different in LMICs, where small bowel perforations are usually due to typhoid fever (TF). TF remains endemic in Asia, Africa, Latin America, the Caribbean, and Oceania [168]. Ileal perforation as a complication of TF and enteritis are major public health problems in many areas worldwide because of its persistent high morbidity and mortality.

Intestinal perforation is the most serious complication of typhoid fever estimated to be solely responsible for 25% of deaths [168]. In most parts of the world, the perforation rate ranges from 0.6 to 4.9% of enteric fever cases, but in West Africa, higher rates between 10 and 33% have been reported [169].

Typhoid ileal perforations have a mortality rate up to 60% [170]. Typhoid ileal perforations represent the third etiology of peritonitis and the primary cause of death due to peritonitis in LMICs [171].

In the CIAOW study, according to stepwise multivariate analysis, the presence of small bowel perforation was an independent variable predictive of mortality [103]. The most common clinical presentation of enteric perforation is abdominal pain and fever, which typically occurs in the third week of disease. Lack of an incidence database and poor financial resources preclude adequate prevention of this public health menace [168]. Mortality from typhoid ileal perforations has been on gradual but variable decline worldwide. Centers capable of better quality of care are now reporting mortality rates less than 5%. The decline has resulted from improved understanding of the disease pathogenesis and progress in supportive and surgical care [172]. The perforation usually affects 40 cm of the terminal ileum in 72–78% of cases; the jejunum, caecum, colon, and gallbladder are affected to a lesser degree. There are rare reports of combined duodenal and appendiceal perforations. Perforations may be multiple (3–40%) [169].

The preoperative diagnosis of perforation usually is based on findings of peritonitis in a patient with a history of prolonged febrile illness. In a prospective study, 53 consecutive patients with typhoid perforation were surgically treated; the morbidity rate in this series was 49.1%, and the most common post-operative complications included wound infection, wound dehiscence, burst abdomen, residual intra-abdominal abscesses, and entero-cutaneous fistulae. The mortality rate was 15.1% and it was significantly affected by the presence of multiple perforations, severe peritoneal contamination, and burst abdomen [172].

### Diagnosis

#### Clinical signs and symptoms

• Severe, sudden-onset periumbilical pain, which can become generalized

• Abdominal tenderness

• Fever

#### Laboratory markers

• Increased white blood cell

• Leucocyte shift to left (> 75%)

• C-reactive protein

#### Imaging

- US
- CT
- Angiography (if there is suspicion of acute mesenteric ischemia)

The sonographic findings suggestive of small bowel perforation are presence of extra-luminal air, a fluid collections and inflammatory changes adjacent to a thickened small bowel segment. In hemodynamically stable patients, triple contrast CT (oral, rectal and intravenous) using water-soluble contrast is the imaging modality of choice for suspected small bowel perforation. It gives anatomical details of the intestinal wall, detects secondary signs of underlying bowel pathology and surrounding mesentery, and picks up even small amounts of extra-luminal air or oral contrast leakage into the peritoneal cavity. In septic and unstable patients in the ICU with uncertain preoperative diagnosis, bedside diagnostic laparoscopy is being used in diagnosis and decision-making thus shortening the observation period.

#### Treatment

- In patients with sepsis-induced tissue hypoperfusion or septic shock prompt administration of 30 mL/kg intravenous crystalloid fluid. If blood pressure is not restored after initial fluid resuscitation vasopressors should be commenced.

- Open or laparoscopic small bowel segmental resection and primary anastomosis.
- In the setting of perforation due to small bowel ischemia, resection and delayed anastomoses at a second look are usually needed. Also, open or endovascular mesenteric vessel reconstruction may be needed.
- Open or laparoscopic resection and stoma creation or exteriorization of the perforation as a stoma (critically ill patients or severe inflammation and edema of the bowel, resulting in friable tissue which precludes anastomosis).
- In the setting of typhoidal perforation, although closure in two layers of single perforation with relatively healthy tissue after refreshment of the edge seems an acceptable option, resection of the unhealthy tissue segment with primary anastomosis of healthy edges about 10 cm on each side of the perforation is recommended.

+

Antibiotic therapy for 4 days (immunocompetent and stable patients) (more days if there are signs of ongoing infection).

There are many methods of surgical treatment of small bowel perforation, including wedge resection and closure, resection and primary anastomosis, and exteriorization of the perforation as a stoma. Primary repair is only rarely an option, in the occasional patient with minimal peritoneal contamination of the peritoneal cavity and a small puncture hole [172–175].

In delayed cases with diffuse peritonitis, there can be severe inflammation and edema of the bowel, resulting in friable tissue which precludes anastomosis after resection, and therefore, an ileostomy should be performed as a life saving measure [176].

Damage control surgery can be an alternative option deferring the definitive surgical management via anastomosis [177].

Laparoscopic management of small bowel perforations has been reported, but there are no comparative studies with open surgery [178]. In Table 12, the clinical pathway for patients with acute diverticulitis is illustrated.

**Table 12:** Clinical pathway for patients with small bowel perforation is illustrated

Small bowel perforation	
**Clinical signs and symptoms** • Severe, sudden-onset periumbilical pain, which can become generalized • Abdominal tenderness • Fever **Laboratory markers** • Increased white blood cell • Leucocyte shift to left (> 75%) • C-reactive protein **Imaging** • US • CT • Angiography (if there is suspicion of acute mesenteric ischemia)	**Diagnosis**
• Open or laparoscopic small bowel segmental resection and primary anastomosis. • In the setting of perforation due to small bowel ischemia, resection and delayed anastomoses at a second look are usually needed. Also, open or endovascular mesenteric vessel reconstruction may be needed. • Open or laparoscopic resection and stoma creation or exteriorization of the perforation as a stoma (critically ill patients or severe inflammation and edema of the bowel, resulting in friable tissue which precludes anastomosis). • In the setting of typhoidal perforation, although closure in two layers of single perforation with relatively healthy tissue after refreshment of the edge seems an acceptable option, resection of the unhealthy tissue segment with primary anastomosis of healthy edges about 10 cm on each side of the perforation is recommended. + Antibiotic therapy for 4 days (immunocompetent and stable patients) (more days if there are signs of ongoing infection). In patients with sepsis-induced tissue hypoperfusion or septic shock prompt administration of 30 mL/kg intravenous crystalloid fluid. If blood pressure is not restored after initial fluid resuscitation vasopressors should be commenced.	**Treatment**
Amoxicillin/clavulanate 2.2 g every 8 h +/− gentamicin 5–7 mg/Kg every 24 h *Avoid Amoxicillin/clavulanate if local Enterobacteriaceae resistances > 20%.* Piperacillin/tazobactam 6 g/0.75 g LD then 4 g/0.5 g every 6 h or 16 g/2 g by continuous infusion +/− Gentamicin 5–7 mg/Kg every 24 h (in critically ill patients) Ceftriaxone 2 g every 24 h + metronidazole 500 mg every 8 h Cefotaxime 2 g every 8 h + metronidazole 500 mg every 8 h or In patients with beta-lactam allergy A fluoroquinolone-based regimen Ciprofloxacin 400 mg every 8/12 h + metronidazole 500 mg every 8 h Or or An aminoglycoside-based regimen Amikacin 15-20 mg/kg every 24 h + metronidazole 500 mg every 8 h or In patients at high risk for infection with community-acquired ESBL-producing Enterobacteriaceae One of the following antibiotics Tigecycline 100 mg LD, then 50 mg every 12 h (carbapenem-sparing strategy) Ertapenem 1 g every 24 h Meropenem 1 g every 8 h (only in patients with septic shock) Doripenem 500 mg every 8 h (only in patients with septic shock) Imipenem/cilastatin 500 mg every 6 h (only in patients with septic shock) In patients at high risk for infection from Enterococci including immunocompromised patients or patients with recent antibiotic exposure, consider the use of ampicillin 2 g every 6 h if patients are not being treated with piperacillin/tazobactam or imipenem/cilastatin (active against ampicillin-susceptible enterococci) or tigecycline.	**Antibiotic therapy**

#### Antibiotic treatment

*Empiric antibiotic regimens suggested. Normal renal function*

One of the following intravenous antibiotics

Amoxicillin/clavulanate 2.2 g every 8 h

*Increasing rates of antimicrobial resistance to amoxicillin/clavulanate among E. coli and other Enterobacteriaceae worldwide, during the last decade, has compromised clinical utility of this agent for empirical therapy of serious Gram-negative infections and therefore it should be used based on local rates of resistance. Avoid its use if Enterobacteriaceae resistances > 20%.*

Piperacillin/tazobactam loading dose 6 g/0.75 g then 4 g/0.5 g every 6 h or 16 g/2 g by continuous infusion, (in critically ill patients)

Ceftriaxone 2 g every 24 h + metronidazole 500 mg every 8 h

Cefotaxime 2 g every 8 h + metronidazole 500 mg every 8 h

or

In patients with beta-lactam allergy

A fluoroquinolone-based regimen

Ciprofloxacin 400 mg every 8 or 12 h + metronidazole 500 mg every 8 h

or

An aminoglycoside-based regimen

Amikacin 15–20 mg/kg every 24 h + metronidazole 500 mg every 8 h

or

In patients at high risk for infection with community-acquired ESBL-producing Enterobacteriaceae

One of the following intravenous antibiotics

Tigecycline 100 mg LD, then 50 mg every 12 h (carbapenem-sparing strategy)

Ertapenem 1 g every 24 h

Meropenem 1 g every 8 h (only in patients with septic shock)

Doripenem 500 mg every 8 h (only in patients with septic shock)

Imipenem/cilastatin 500 mg every 6 h (only in patients with septic shock)

In patients at high risk for infection from enterococci including immunocompromised patients or patients with recent antibiotic exposure, consider the use of ampicillin 2 g every 6 h if patients are not being treated with piperacillin/tazobactam or imipenem/cilastatin (active against ampicillin-susceptible enterococci) or tigecycline.

## Gastroduodenal ulcer perforation

Gastroduodenal perforations may be spontaneous or traumatic and most of the spontaneous perforations are due to peptic ulcer disease. Improved medical management of peptic ulceration has reduced the incidence of perforation, but still remains a common cause of peritonitis. Chichom-Mefire et al. in 2016 reported gastroduodenal perforations as the leading cause of peritonitis in the tropics [171]. The majority of perforated peptic ulcers are caused by *Helicobacter pylori*, so apart from simple closure, definitive surgery is not usually required. Perforated peptic ulcer is an indication for operation in nearly all cases except when the patient is unfit for surgery.

Gastroduodenal ulcer perforations have decreased in frequency in the last few years, largely due to the widespread adoption of medical therapies for peptic ulcer disease and decreasing incidence of *Helicobacter pylori* infection in Western countries. However, ulcer disease is still a common emergency condition worldwide and is associated with mortality rates of up to 30% [179]. The main etiologic factors include the use of non-steroidal anti-inflammatory drugs (NSAIDs), steroids, smoking, *Helicobacter pylori*, and a diet high in salt. All these factors have in common that they affect acid secretion and impairment of the gastric mucosal protection [180]. Stress ulcers with perforation may occur in critically ill patients in intensive care, where the diagnosis may be obscured owing to the lack of signs and symptoms in an unconscious or sedated patient, highlighting the role of prophylaxis in these patients.

### Diagnosis

#### Clinical signs and symptoms

- Severe, sudden-onset epigastric pain, which can become generalized
- Abdominal tenderness
- Fever
- Abdominal distension, tenderness, and rigidity with masked liver dullness and absent bowel sounds

#### Laboratory markers

- White blood cell count
- Leucocyte left shift (>75%)
- C-reactive protein

#### Imaging

- CT
- Plain abdominal x-ray
- US

#### Imaging findings

Signs of gastrointestinal perforation (extraluminal gas, intra-abdominal fluid)

Air pockets around the stomach and duodenum and thick reactive intestinal wall

### Treatment

- Laparoscopic/open simple or double-layer suture with or without an omental patch is a safe and effective procedure to address small perforated ulcers (standard procedure).
- Distal gastrectomy (large perforations near the pylorus; suspicion of malignancy).

+

Antibiotic therapy for 4 days (immunocompetent and stable patients) (more days if there are signs of ongoing sepsis).

Surgery is the most effective means of source control in patients with gastroduodenal perforation [181]. A perforated gastric ulcer needs careful assessment. A proportion (9%) will be malignant [182] and gastric ulcers are more likely to re-perforate after simple closure with high mortality (15%) [182]. Tissue biopsies from the edge of the ulcer are taken because of the risk of malignancy, even in a benign-looking condition. The main surgical treatment for peptic ulcer perforation has become a simple suture of the perforation site with or without the addition of an omental patch [183].

In 2010, Lo et al. conducted a study to determine if an omental patch offers any clinical benefit that is not offered by simple closure alone [184]. The study demonstrated that, in terms of leakage rates and overall surgical outcome, covering the repaired perforated peptic ulcer with an omental patch did not convey additional advantages compared to simple closure alone.

Scoring systems to predict disease severity or outcome in patients with gastroduodenal perforations are unreliable and not accurate and cannot be generalized from one population to another [185]. The closure of ‘healthy’ gastric tissue, as well as providing histology, is the goal. However, a distal gastrectomy should be considered if the closure of a larger perforation is difficult and/or in case of suspicion of malignancy, the patient is sufficiently fit and the surgeon sufficiently experienced. Chung et al. [186] noted that less than 10% of perforated peptic ulcer (PPU) patients required gastric resection and with a mortality risk of 24% the outcome was more inferior than omental patch repair.

Laparoscopic repair of PPU is a safe and effective procedure in experienced hands. The literature was summarized in a recent systematic review [187]. The authors concluded that laparoscopic surgery results are not clinically different from those of open surgery. Further data is required to investigate the potentially long learning curve seen among participating surgeons.

Conservative treatment for PPU is seldom reported and restricted mostly to case reports and series of patients that are critically ill and unsuitable for operative intervention [188].

In Table 13, the clinical pathway for patients with gastroduodenal perforation is illustrated.

**Table 13:** clinical pathway for patients with gastroduodenal perforation is illustrated

Gastroduodenal perforation	
**Clinical signs and symptoms** • Severe, sudden-onset epigastric pain, which can become generalized • Abdominal tenderness • Fever • Abdominal distension, tenderness, and rigidity with masked liver dullness and absent bowel sounds **Laboratory markers** • White blood cell count • Leucocyte left shift (> 75%) • C-reactive protein **Imaging** • CT • Plain abdominal x-ray • US	**Diagnosis**
• Laparoscopic/open simple or double-layer suture with or without an omental patch is a safe and effective procedure to address small perforated ulcers (standard procedure). • Distal gastrectomy (large perforations near the pylorus; suspicion of malignancy). + Antibiotic therapy for 4 days (immunocompetent and stable patients) (more days if there are signs of ongoing sepsis).	**Treatment**
Amoxicillin/clavulanate 2.2 g every 8 h +/− gentamicin 5–7 mg/Kg every 24 h *Avoid Amoxicillin/clavulanate if local Enterobacteriaceae resistances > 20%.* Piperacillin/tazobactam 6 g/0.75 g LD then 4 g/0.5 g every 6 h or 16 g/2 g by continuous infusion +/− gentamicin 5–7 mg/Kg every 24 h (in critically ill patients) Ceftriaxone 2 g every 24 h + metronidazole 500 mg every 8 h Cefotaxime 2 g every 8 h + metronidazole 500 mg every 8 h or In patients with beta-lactam allergy A fluoroquinolone-based regimen Ciprofloxacin 400 mg every 8/12 h + metronidazole 500 mg every 8 h or An aminoglycoside-based regimen Amikacin 15–20 mg/kg every 24 h + metronidazole 500 mg every 8 h	**Antibiotic therapy**

#### Antibiotic treatment

*Empiric antibiotic regimens. Normal renal function*

One of the following intravenous antibiotics

Amoxicillin/clavulanate 2.2 g every 8 h

*Increasing rates of antimicrobial resistance to amoxicillin/clavulanate among E. coli and other Enterobacteriaceae worldwide, during the last decade, has compromised clinical utility of this agent for empirical therapy of serious Gram-negative infections and therefore it should be used based on local rates of resistance. Avoid its use if Enterobacteriaceae resistances > 20%.*

Piperacillin/tazobactam 6 g/0.75 g LD then 4 g/0.5 g every 6 h or 16 g/2 g by continuous infusion, (in critically ill patients)

Ceftriaxone 2 g every 24 h + metronidazole 500 mg every 8 h

Cefotaxime 2 g every 8 h + metronidazole 500 mg every 8 h

or

In patients with beta-lactam allergy

A fluoroquinolone-based regimen

Ciprofloxacin 400 mg every 8 or 12 h + metronidazole 500 mg every 8 h

or

An aminoglycoside-based regimen

Amikacin 15–20 mg/kg every 24 h + metronidazole 500 mg every 8 h

## Post-operative peritonitis

Post-operative peritonitis (PP) is a life-threatening hospital-acquired intra-abdominal infection with high mortality rates [189, 190]. The most common cause of PP is an anastomotic leakage [191]. It is most frequent after rectal resection [192, 193], but it may complicate any gastrointestinal anastomosis. Treating patients with post-operative peritonitis requires supportive therapy of organ dysfunction, source control of infection, and intensive antimicrobial therapy. The diagnosis of PP may be difficult because there are no absolute specific clinical signs and laboratory tests to reject or confirm the diagnosis. The atypical clinical presentation may be responsible for a delay in diagnosis and re-intervention or reoperation.

Several recent studies have investigated the role of CRP as an early marker of anastomotic leakage following colorectal surgery. A systematic literature search to identify studies evaluating the diagnostic accuracy of postoperative CRP for anastomotic leakage following colorectal surgery was published by Singh et al. [194]. CRP resulted in a useful negative predictive test for the development of anastomotic leakage following colorectal surgery.

### Diagnosis

#### Clinical signs and symptoms

• Fever

• Abdominal pain

• Abdominal tenderness

#### Laboratory markers

• Increased white blood cell count

• C-reactive protein

• PCT

**Imaging**

• CT

#### Imaging findings

- Signs of intestinal perforation such as extraluminal air bubbles, intra-abdominal fluid
- Post-operative abscess

### Treatment

#### Localized abscess

- Percutaneous drainage and antibiotic therapy.

Antibiotics and drainage may be the optimal means of treating post-operative localized intra-abdominal abscesses in stable patients when there are no signs of generalized peritonitis. Several retrospective studies in the fields of surgery and radiology have documented the effectiveness of percutaneous drainage in the treatment of post-operative localized intra-abdominal abscesses [190].

#### Diffuse peritonitis

- Early surgical source control and antibiotic therapy.

The inability to control the septic source is associated with an intolerably high mortality rate. Organ failure and/or delay in subsequent re-laparotomies which have been delayed for more than 24 h, both result in higher mortality rates in patients affected by post-operative IAIs [195]. Early re-laparotomy appears to be the most effective means of treating post-operative peritonitis [196].

In 2009, a retrospective study by Chichom-Mefire et al. [197] analyzed aspects of re-operative abdominal surgery in an economically disadvantaged environment with respect to indications, operative findings, treatment modalities, and outcomes. Mortality in this series was 18%, increasingly significant when the initial operative procedure was for peritonitis and re-operation was due to septic complications. Operative re-intervention based on clinical findings was considered the favored strategy. In the Western world, reoperation for postoperative abdominal sepsis is ideally based on CT imaging. If as a rule indication for surgery is based on clinical and laboratory findings only, the rate of unnecessary reoperations would be too high and the possibility of treatment of abscesses by percutaneous drainage would be missed too often. US should not be performed as initial diagnostic test in patients with suspicion of postoperative intra-abdominal infection because of its low discriminatory power [198].

In Table 14, the clinical pathway for patients with post-operative peritonitis is illustrated.

**Table 14:** Clinical pathway for patients with acute appendicitis is illustrated

Post-operative peritonitis	
**Clinical signs and symptoms** • Fever • Abdominal pain • Abdominal tenderness **Laboratory markers** • Increased white blood cell • C-reactive protein • PCT **Imaging** • CT	**Diagnosis**
**Localised abscess** • Percutaneous drainage and antibiotic therapy. Antibiotics and drainage may be the optimal means of treating post-operative localized intra-abdominal abscesses in stable patients when there are no signs of generalized peritonitis. • **Diffuse peritonitis** • Early surgical source control and antibiotic therapy. The inability to control the septic source is associated with an intolerably high mortality rate. + Antibiotic therapy	**Treatment**
In patients with no risk for multidrug-resistant organism One of the following intravenous antibiotics Piperacillin/tazobactam 6 g/0.75 g LD then 4 g/0.5 g every 6 h or 16 g/2 g by continuous infusion Tigecycline 100 mg initial dose, then 50 mg every 12 h (carbapenem sparing strategy) Meropenem 1 g every 8 h +/− ampicillin 2 g every 6 h (critically ill patients) Doripenem 500 mg every 8 h +/− ampicillin 2 g every 6 h (critically ill patients) Imipenem/cilastatin 500 mg every 6 h (critically ill patients) +/− In patients at high risk for invasive candidiasis Fluconazole 800 mg LD then 400 mg every 24 h In patients with documented beta-lactam allergy, consider the use of antibiotic combinations with Amikacin 15–20 mg/kg every 24 h In patients with high risk for multidrug-resistant organism One of the following intravenous antibiotics Tigecycline 100 mg LD, then 50 mg every 12 h (not active against Pseudomonas aeruginosa) Eravacycline 1 mg/kg every 12 h (not active against Pseudomonas aeruginosa) + Piperacillin/tazobactam 4.5 every 6 h or In critically ill patients one of the following intravenous antibiotics Meropenem 1 g every 8 h Doripenem 500 mg every 8 h Imipenem/cilastatin 500 mg every 6 h + One of the following intravenous antibiotics Vancomycin 25–30 mg/kg LD then 15–20 mg/kg/dose every 8 h Teicoplanin 12 mg/kg every 12 h 3 LDs then 12 mg/kg every 24 h +/− In patients with high risk for invasive candidiasis In stable patients Fluconazole 800 mg LD then 400 mg every 24 h In unstable patients one of the following antifungal agents Caspofungin 70 mg LD, then 50 mg daily Anidulafungin 200 mg LD, then 100 mg daily Micafungin 100 mg daily Amphotericin B Liposomal 3 mg/kg daily (renal toxicity risk) In patients with suspected or proven infection with MDR (non-metallo-beta-lactamase-producing) Pseudomonas aeruginosa, consider the use of antibiotic combinations with Ceftolozane/tazobactam. In patients with suspected or proven infection with carbapenemase-producing *Klebsiella pneumoniae* and MDR (non-metallo-beta-lactamase-producing) *Pseudomonas aeruginosa*, consider the use of antibiotic combinations with Ceftazidime/Avibactam. In patients with suspected or proven infection with vancomycin-resistant enterococci (VRE) including patients with previous enterococcal infection or colonization, immunocompromised patients, patients with long ICU stay, or recent Vancomycin exposure One of the following intravenous antibiotics Tigecycline 100 mg LD, then 50 mg every 12 h Linezolid 600 mg every 12 h In patients with documented beta-lactam allergy, consider the use of antibiotic combinations with Amikacin 15–20 mg/kg daily.	**Antibiotic therapy**

#### Antibiotic treatment

*Empiric antibiotic regimens. Normal renal function*

In patients with no risk for multidrug-resistant organism

One of the following intravenous antibiotics

Piperacillin/tazobactam 6 g/0.75 g LD then 4 g/0.5 g every 6 h or 16 g/2

g by continuous infusion

Tigecycline 100 mg initial dose, then 50 mg every 12 h (carbapenem-sparing strategy)

Meropenem 1 g every 8 h +/− ampicillin 2 g every 6 h (critically ill patients)

Doripenem 500 mg every 8 h +/− ampicillin 2 g every 6 h (critically ill patients)

Imipenem/cilastatin 500 mg every 6 h (critically ill patients)

+/−

In patients at high risk for invasive candidiasis

Fluconazole 800 mg LD then 400 mg every 24 h

In patients with documented beta-lactam allergy, consider the use of antibiotic combinations with Amikacin 15–20 mg/kg every 24 h

In patients with high risk for multidrug-resistant organism

One of the following intravenous antibiotics

Tigecycline 100 mg LD, then 50 mg every 12 h (not active against Pseudomonas aeruginosa)

Eravacycline 1 mg/kg every 12 h (not active against Pseudomonas aeruginosa)

+

Piperacillin/tazobactam 4.5 every 6 h

or

In critically ill patients

one of the following intravenous antibiotics

Meropenem 1 g every 8 h

Doripenem 500 mg every 8 h

Imipenem/cilastatin 500 mg every 6 h

+

One of the following intravenous antibiotics

Vancomycin 25–30 mg/kg LD then 15–20 mg/kg/dose every 8 h

Teicoplanin 12 mg/kg every 12 h 3 LDs then 12 mg/kg every 24 h

+/−

In patients with high risk for invasive candidiasis

In stable patients

Fluconazole 800 mg LD then 400 mg every 24 h

In unstable patients

one of the following antifungal agents

Caspofungin 70 mg LD, then 50 mg daily

Anidulafungin 200 mg LD, then 100 mg daily

Micafungin 100 mg daily

Amphotericin B Liposomal 3 mg/kg daily (renal toxicity risk)

In patients with suspected or proven infection with MDR (non-metallo-beta-lactamase-producing) *Pseudomonas aeruginosa*, consider the use of antibiotic combinations with ceftolozane/tazobactam.

In patients with suspected or proven infection with carbapenemase-producing *Klebsiella pneumoniae* and MDR (non-metallo-beta-lactamase-producing) *Pseudomonas aeruginosa*, consider the use of antibiotic combinations with Ceftazidime/Avibactam.

In patients with suspected or proven infection with vancomycin-resistant enterococci (VRE) including patients with previous enterococcal infection or colonization, immunocompromised patients, patients with long ICU stay, or recent Vancomycin exposure

One of the following intravenous antibiotics

Tigecycline 100 mg LD, then 50 mg every 12 h

Linezolid 600 mg every 12 h

In patients with documented beta-lactam allergy, consider the use of antibiotic combinations with Amikacin 15–20 mg/kg daily.

## Post-traumatic perforation

Trauma continues to be a major public health problem worldwide, and it is associated with high morbidity and mortality regardless of the socioeconomic status [199]. Both blunt and penetrating mechsnisms may result in bowel injury. Motor vehicle crashes remain the most common and falls the next most common cause of blunt force trauma globally [200].

Hollow viscus injury (HVI) has a more insidious presentation in this setting, often resulting in delayed diagnosis. Clinical signs may take time to develop, and imaging investigations are not completely sensitive. In addition, other distracting injuries may affect an accurate and timely diagnosis. Improved outcome is reported in settings where, availability of advanced imaging modalities, patient monitoring capabilities, and prompt intervention are possible, whereas limited diagnostic capabilities, late presentation, and late intervention adversely affect outcomes [201, 202]. Several mechanisms of bowel injury have been documented in the wake of blunt abdominal trauma. The most common injury is either posterior crushing of the bowel segment between the seat belt and a vertebral body or pelvis or a shearing insult during deacceleration across the edge of a seat belt or other object. These can result in local lacerations of the bowel wall, mural and mesenteric hematomas, transection of the bowel, localized devascularization, and full-thickness contusions. Devitalization of the areas of contusion may subsequently result in late perforation. Colonic injuries appear to occur less frequently than small intestine injuries perhaps due to its location and the lack of redundancy, which prevents the formation of closed loops. When colonic injury does occur it is frequently in the sigmoid colon and cecum where shear is caused by lap-only seat belts. Abdominal trauma may be associated with other additional co-morbid injuries, which could complicate the management and affect the outcome. Delay in diagnosis and treatment of the HVI may result in early peritonitis, hemodynamic instability and increased mortality and morbidity.

### Diagnosis

#### Clinical signs and symptoms

• Fever

• Abdominal pain

• Abdominal tenderness

#### Laboratory markers

• Increased of white blood cell count

• C-reactive protein

• PCT

#### Imaging

• CT

• US

#### Imaging findings

- Signs of intestinal perforation (extraluminal gas, intra-abdominal fluid)

Focused Assessment with Sonography in Trauma (FAST) is an initial step in assessment of hemodynamically unstable patients with blunt abdominal injury. It primarily detects the presence of free fluid but identifies free air only in 8% of the cases with traumatic bowel perforation. Abdominal CT scan is performed in hemodynamically stable patients and the findings considered diagnostic for bowel perforation are contrast extravasation and/or extra-luminal air. Fluid between the mesentery, thickening of bowel wall are also common CT signs.

More subtle but important concerns for injury is the presence of thickened segments of bowel wall, associated mesenteric hematomas and free fluid in the pelvis without evidence of a solid organ injury consistent with a bleeding source. In the current approach of non-operative management (NOM) of stable patients with solid organ injury, the presence of fluid without solid organ injury should warrant consideration for laparotomy/laparoscopy to evaluate for possible visceral perforation.

### Treatment

- Open/laparoscopic surgical repair. Use of laparoscopy in blunt trauma is highly debatable.
- Resection and primary anastomosis.
- Stoma (in critically ill patients and/or colorectal injuries involving all layers in the setting of multiple injuries).

+

Perioperative antibiotics. If hollow viscus injury is repaired within 12 h, antibiotics should be continued for ≤ 24 h

Early clinical recognition and surgical intervention is important in case of HVI [202, 204, 205].

Repair or anastomosis of intestinal injuries should be considered in all patients. A complete diversion of the fecal stream should be considered in colorectal injuries involving all layers in the setting of multiple injuries and resultant physiological compromise, unfavorable comorbid conditions, and perhaps in the setting of delayed diagnoses [206]. Damage control laparotomy (DCL) in the context of HVI is accepted for small bowel injury in the context of coagulopathy, while temporary colon ligation and non-continuity has been debated because of potential complications and an increased incidence of leakage. However, delayed anastomosis of colon injuries after DCL has been increasingly advocated and in trauma and shown to safely avoid unnecessary stoma creation in most patients who are not candidates for anastomosis during initial intervention [206]. If primary fascial closure is not possible, a negative pressure-based temporary closure device should be used for progressive closure steps over the next days.

If hollow viscus injury is repaired within 12 h, antibiotics should be continued for ≤ 24 h [207].

In Table 15, the clinical pathway for patients with post-traumatic perforation is illustrated.

**Table 15:** Clinical pathway for patients with post-traumatic is illustrated

Post-traumatic peritonitis	
**Clinical signs and symptoms** • Fever • Abdominal pain • Abdominal tenderness **Laboratory markers** • Increased white blood cell • C-reactive protein • PCT **Imaging** • CT • US	**Diagnosis**
• Open/laparoscopic surgical repair. Use of laparoscopy in blunt trauma is highly debatable • Resection and primary anastomosis • Stoma (in critically ill patients and/or colorectal injuries involving all layers in the setting of multiple injuries). + Perioperative antibiotics. If hollow viscus injury is repaired within 12 h, antibiotics should be continued for ≤ 24 h	**Treatment**
Amoxicillin/clavulanate 2.2 g every 8 h +/− gentamicin 5-7 mg/Kg every 24 h *Avoid Amoxicillin/clavulanate if local Enterobacteriaceae resistances > 20%.* Piperacillin/tazobactam 6 g/0.75 g LD then 4 g/0.5 g every 6 h or 16 g/2 g by continuous infusion +/− gentamicin 5–7 mg/Kg every 24 h (in critically ill patients) Ceftriaxone 2 g every 24 h + metronidazole 500 mg every 8 h Cefotaxime 2 g every 8 h + metronidazole 500 mg every 8 h or In patients with beta-lactam allergy A fluoroquinolone-based regimen Ciprofloxacin 400 mg every 8/12 h + metronidazole 500 mg every 8 h or An aminoglycoside regimen Amikacin 15-20 mg/kg every 24 h + metronidazole 500 mg every 8 h or In patients at high risk for infection with community-acquired ESBL-producing Enterobacteriaceae One of the following antibiotics Tigecycline 100 mg LD, then 50 mg every 12 h (carbapenem-sparing strategy) Ertapenem 1 g every 24 h Meropenem 1 g every 8 h (only in patients with septic shock) Doripenem 500 mg every 8 h (only in patients with septic shock) Imipenem/cilastatin 500 mg every 6 h (only in patients with septic shock) In patients at high risk for infection with Enterococci including immunocompromised patients or patients with recent antibiotic exposure, consider the use of ampicillin 2 g every 6 h if patients are being treated with ertapenem/meropenem or doripenem	**Antibiotic therapy**

#### Antibiotic treatment

*Empiric antibiotic regimens. Normal renal function*

One of the following intravenous antibiotics

Amoxicillin/clavulanate 2.2 g every 8 h

*Increasing rates of antimicrobial resistance to amoxicillin/clavulanate among E. coli and other Enterobacteriaceae worldwide, during the last decade, has compromised clinical utility of this agent for empirical therapy of serious Gram-negative infections and therefore it should be used based on local rates of resistance. Avoid its use if Enterobacteriaceae resistances > 20%.*

Piperacillin/tazobactam 6 g/0.75 g LD then 4 g/0.5 g every 6 h or 16 g/2 g by continuous infusion, (in critically ill patients)

Ceftriaxone 2 g every 24 h + metronidazole 500 mg every 8 h

Cefotaxime 2 g every 8 h + metronidazole 500 mg every 8 h

or

In patients with beta-lactam allergy

A fluoroquinolone-based regimen

Ciprofloxacin 400 mg every 8 or12 h + metronidazole 500 mg every 8 h

or

An aminoglycoside regimen

Amikacin 15-20 mg/kg every 24 h + metronidazole 500 mg every 8 h

or

In patients at high risk for infection with community-acquired ESBL-producing Enterobacteriaceae

One of the following antibiotics

Tigecycline 100 mg LD, then 50 mg every 12 h (carbapenem-sparing strategy)

Ertapenem 1 g every 24 h

Meropenem 1 g every 8 h (only in patients with septic shock)

Doripenem 500 mg every 8 h (only in patients with septic shock)

Imipenem/cilastatin 500 mg every 6 h (only in patients with septic shock)

In patients at high risk for infection with Enterococci including immunocompromised patients or patients with recent antibiotic exposure, consider the use of ampicillin 2 g every 6 h if patients are being treated with ertapenem/ meropenem or doripenem.

## Tertiary peritonitis (ongoing peritonitis)

Tertiary peritonitis is an infection of the peritoneal cavity that occurs after seemingly successful surgical source control of secondary peritonitis. Actually, complete recovery from secondary peritonitis, however, has not been achieved. It is more common among critically ill or immunocompromised patients and often associated with multidrug-resistant organisms (MDROs). It is typically associated with high morbidity and mortality. Tertiary peritonitis was previously seen as a distinct entity, but essentially it represents an evolution and complication of secondary peritonitis [170, 208, 209]. The term “ongoing peritonitis” [210] or “persistent peritonitis” [211] may better indicate that it is not a different disease to secondary peritonitis, but rather represents secondary peritonitis lasting longer and harboring other (selected and more resistant) pathogens.

### Diagnosis

#### Laboratory markers

- Increased of white blood cell count
- C-reactive protein
- PCT

#### Imaging

- CT

### Treatment

- On-demand re-laparotomy and/or antibiotic therapy

In patients who are prone to persistent infections regardless of eradication of the source of infection timely relaparotomy provides the only surgical option that significantly improves outcome. In these cases, single operation may not be sufficient to achieve source control; thus, relaparotomy may become necessary [63, 212, 213]. Re-laparotomy on demand is the treatment of choice [32].

The benefits and potential harms of an OA has been discussed in the ‘principles of source control’.

Initial severity, the presence of *Candida* spp*.* in surgical samples and inadequate source control have been seen the major risk factors for persistent peritonitis. Montravers et al. [211] demonstrated a progressive shift of peritoneal flora with the number of reoperations, comprising extinction of susceptible strains and emergence of both Gram negative and Gram positive multidrug-resistant strains and fungi. Emergence of multidrug-resistant bacteria was frequent and increases progressively directly with the number of reoperations. Surprisingly, no link was demonstrated between emergence of multidrug-resistant strains and specific antibiotic regimens, while source control and its timing appeared to be major determinants of the emergence of multidrug-resistant strains. A better understanding of intra-abdominal infections and their management requires additional tools to distinguish colonizsing organisms from infective pathogenic organisms in order to determine which organisms should need to be treated. In Table 16, the clinical pathway for patients with tertiary (ongoing) peritonitis is illustrated.

**Table 16:** Clinical pathway for patients with tertiary (ongoing peritonitis) is illustrated

Tertiary peritonitis	
**Clinical signs and symptoms** • Fever • Abdominal pain • Abdominal tenderness **Laboratory markers** • Increased white blood cell • C-reactive protein • PCT **Imaging** • CT	**Diagnosis**
• On-demand re-laparotomy and/or antibiotic therapy	**Treatment**
In patients with no risk for multidrug-resistant organism One of the following intravenous antibiotics Piperacillin/tazobactam 6 g/0.75 g LD then 4 g/0.5 g every 6 h or 16 g/2 g by continuous infusion Tigecycline 100 mg initial dose, then 50 mg every 12 h (carbapenem-sparing strategy) Meropenem 1 g every 8 h +/− ampicillin 2 g every 6 h (critically ill patients) Doripenem 500 mg every 8 h +/− ampicillin 2 g every 6 h (critically ill patients) Imipenem/cilastatin 500 mg every 6 h (critically ill patients) +/− In patients at high risk for invasive candidiasis Fluconazole 800 mg LD then 400 mg every 24 h In patients with documented beta-lactam allergy, consider the use of antibiotic combinations with Amikacin 15–20 mg/kg every 24 h In patients with high risk for multidrug-resistant organism One of the following intravenous antibiotics Tigecycline 100 mg LD, then 50 mg every 12 h (not active against *Pseudomonas aeruginosa*) Eravacycline 1 mg/kg every 12 h (not active against *Pseudomonas aeruginosa*) + Piperacillin/tazobactam 4.5 every 6 h or In critically ill patients one of the following intravenous antibiotics Meropenem 1 g every 8 h Doripenem 500 mg every 8 h Imipenem/cilastatin 500 mg every 6 h + One of the following intravenous antibiotics Vancomycin 25–30 mg/kg LD then 15–20 mg/kg/dose every 8 h Teicoplanin 12 mg/kg every 12 h 3 LDs then 12 mg/kg every 24 h +/− In patients with high risk for invasive candidiasis In stable patients Fluconazole 800 mg LD then 400 mg every 24 h In unstable patients one of the following antifungal agents Caspofungin 70 mg LD, then 50 mg daily Anidulafungin 200 mg LD, then 100 mg daily Micafungin 100 mg daily Amphotericin B Liposomal 3 mg/kg daily In patients with suspected or proven infection with MDR (non-metallo-beta-lactamase-producing) *Pseudomonas aeruginosa*, consider the use of antibiotic combinations with ceftolozane/tazobactam. In patients with suspected or proven infection with carbapenemase-producing *Klebsiella pneumoniae* and MDR (non-metallo-beta-lactamase-producing) *Pseudomonas aeruginosa*, consider the use of antibiotic combinations with Ceftazidime/Avibactam. In patients with suspected or proven infection with vancomycin-resistant enterococci (VRE) including patients with previous enterococcal infection or colonization, immunocompromised patients, patients with long ICU stay, or recent Vancomycin exposure One of the following intravenous antibiotics Tigecycline 100 mg LD, then 50 mg every 12 h Linezolid 600 mg every 12 h In patients with documented beta-lactam allergy, consider the use of antibiotic combinations with Amikacin 15–20 mg/kg daily.	**Antibiotic therapy**

#### Antibiotic treatment

*Empiric antibiotic regimens. Normal renal function*

One of the following intravenous antibiotics

Tigecycline 100 mg LD, then 50 mg every 12 h (not active against *Pseudomonas aeruginosa*)

Eravacycline 1 mg/kg every 12 h (not active against *Pseudomonas aeruginosa*)

+

Piperacillin/tazobactam 4.5 every 6 h

or

In critically ill patients

one of the following intravenous antibiotics

Meropenem 1 g every 8 h

Doripenem 500 mg every 8 h

Imipenem/cilastatin 500 mg every 6 h

+

One of the following intravenous antibiotics

Vancomycin 25–30 mg/kg LD then 15–20 mg/kg/dose every 8 h

Teicoplanin 12 mg/kg every 12 h 3 LDs then 12 mg/kg every 24 h

+/−

In patients with high risk for invasive candidiasis

In stable patients

Fluconazole 800 mg LD then 400 mg every 24 h

In unstable patients

one of the following antifungal agents

Caspofungin 70 mg LD, then 50 mg daily

Anidulafungin 200 mg LD, then 100 mg daily

Micafungin 100 mg daily

Amphotericin B Liposomal 3 mg/kg daily

In patients with high risk for multidrug-resistant organism

One of the following intravenous antibiotics

Tigecycline 100 mg LD, then 50 mg every 12 h (No active against Pseudomonas aeruginosa)

Eravacycline 1 mg/kg every 12 h (No active against Pseudomonas aeruginosa)

+

Piperacillin/tazobactam 4.5 every 6 h

or

In critically ill patients

one of the following intravenous antibiotics

Meropenem 1 g every 8 h

Doripenem 500 mg every 8 h

Imipenem/cilastatin 500 mg every 6 h

+

One of the following intravenous antibiotics

Vancomycin 25–30 mg/kg loading dose then 15–20 mg/kg/dose every 8 h

Teicoplanin 12 mg/kg every 12 h times 3 loading doses then 12 mg/kg every 24 h

+/−

In patients with high risk for invasive candidiasis

In stable patients

Fluconazole 800 mg LD then 400 mg every 24 h

In unstable patients

one of the following antifungal agents

Caspofungin 70 mg LD, then 50 mg daily

Anidulafungin 200 mg LD, then 100 mg daily

Micafungin 100 mg daily

Amphotericin B Liposomal 3 mg/kg daily (significant renal toxicity risk)

In patients with suspected or proven infection with MDR (non-metallo-beta-lactamase-producing) *Pseudomonas aeruginosa*, consider the use of antibiotic combinations with ceftolozane/tazobactam.

In patients with suspected or proven infection with carbapenemase-producing *Klebsiella pneumoniae* and MDR (non-metallo-beta-lactamase-producing) *Pseudomonas aeruginosa*, consider the use of antibiotic combinations with Ceftazidime/Avibactam.

In patients with suspected or proven infection with vancomycin-resistant enterococci (VRE) including patients with previous enterococcal infection or colonization, immunocompromised patients, patients with long ICU stay, or recent Vancomycin exposure

One of the following intravenous antibiotics

Tigecycline 100 mg LD, then 50 mg every 12 h

Linezolid 600 mg every 12 h

In patients with documented beta-lactam allergy, consider the use of antibiotic combinations with Amikacin 15–20 mg/kg daily.

## Conclusion

IAIs are common surgical emergencies and have been reported as major contributors to non-trauma deaths in emergency departments worldwide. The cornerstones of effective treatment of IAIs include early recognition, adequate source control, appropriate antimicrobial therapy, and prompt physiologic stabilization in critically ill patients. Affecting both high-income and low and middle-income countries, IAIs are a tremendous source of lost life, livelihood, and resources.

This document is presented in light of the aim to facilitate clinical management of IAIs worldwide building evidence-based clinical pathways for the most common IAIs.

## Data Availability

Not applicable

## Abbreviations

IAI: Intra-abdominal infection
MDR: Multi-drug-resistant
ESBLs: Extended-spectrum beta-lactamases
